# Enhancing crop sustainability: GSEBNet for precise tomato leaf disease classification

**DOI:** 10.3389/fpls.2026.1947340

**Published:** 2026-10-28

**Authors:** Muhammad Sheraz Khan, Irshad Ahmad, Kainat Nisa, Faisal Alharbi, Mohammed Aloraini, Shabana Habib, Muhammad Islam

**Affiliations:** 1 Department of Computer Science, Islamia College University, Peshawar, Khyber Pakhtunkhwa, Pakistan; 2 Quantum Technologies and Advanced Computing Institute, King Abdulaziz City for Science and Technology, Riyadh, Saudi Arabia; 3 Department of Electrical Engineering, College of Engineering, Qassim University, Buraydah, Saudi Arabia; 4 Department of Information Technology, College of Computer, Qassim University, Buraydah, Saudi Arabia

**Keywords:** classification, CNN, deep learning, residual block, SE blocks, tomato leaf disease, transfer learning

## Abstract

**Introduction:**

Plant diseases pose a major threat to food-supply sustainability. Early identification of crop diseases is crucial for agricultural production and crop yield. Classifying tomato leaves helps determine the treatments needed to keep crops healthy. Although many deep learning approaches have been proposed for plant disease classification, most rely on laboratory-captured images and struggle with real-field conditions.

**Methods:**

This study introduces an extensive class-based dataset collected in natural environments, visually distinguishing 13 infectious diseases and healthy tomato leaves. We propose the Gamma-preprocessed Squeeze-Excitation Block Network (GSEBNet), a lightweight CNN architecture that integrates Squeeze-and-Excitation (SE) blocks with carefully configured Mobile Inverted Bottleneck Convolution (MBConv) layers and a domain-specific Gamma-Unsharp Brightness Reduction (GUBR) preprocessing pipeline. The model was trained and evaluated on a combined dataset of field-captured (PlantCity) and laboratory (PlantVillage) images covering 14 classes.

**Results:**

The proposed GSEBNet achieved a test accuracy of 99.48% on the 14-class dataset, with precision, recall, F1-score, and Cohen’s Kappa of 99.49%, 99.49%, 0.99, and 0.9944, respectively.

**Discussion:**

The obtained results are competitive with those reported in previous studies, while acknowledging differences in datasets and experimental settings. The synergistic co-design of GUBR preprocessing with SE-enhanced MBConv layers significantly improves feature discriminability in low-quality field-captured images.

## 1 Introduction

Global population growth and the intensification of agricultural production have elevated tomato to one of the most economically significant horticultural crops worldwide. The Food and Agriculture Organization of the United Nations (FAO) estimates the total global production of tomatoes to be more than 189 million metric tons annually on an area of about 5 million hectares of cultivated land (Panno et al., 2021). Tomatoes play an important role in food security and income generation in both developed and developing countries, serving as a key raw material for fresh produce and processed products such as sauces, juices, and pastes (Motamedzadegan and Tabarestani, 2018). Tomato is highly susceptible to various biotic stresses, including fungal, bacterial, viral, and pest diseases, which can cause yield losses of 20–80% if not detected (Wspanialy and Moussa, 2020). Tomato is a crop that is consistently affected by diseases like early blight, late blight, leaf mold, septoria leaf spot, bacterial spot, target spot, Fusarium wilt, Verticillium wilt, spider mite damage, tomato mosaic virus (ToMV), tomato yellow leaf curl virus (TYLCV), and leaf miner damage, which are persistent threats to tomato production worldwide (Foolad et al., 2008).

Traditional disease control in tomato production relies heavily on field scouting and the experience of trained agronomists. This is a labor-intensive, time-consuming, and subjective process, and the diagnostic accuracy is limited by the pathologist’s expertise and the phenotypic subtlety of early lesions (Hu et al., 2026). Furthermore, specialized plant pathology services are highly limited in resource-poor environments like smallholder farms in South Asia, including Pakistan, one of the world’s top tomato producers (Khan et al., 2023). Consequently, disease misdiagnosis and lack of timely action frequently lead to high pre-harvest losses and to the indiscriminate use of pesticides, which further aggravates the economic and environmental impact.

With the emergence of deep learning (DL) (LeCun et al., 2015) and computer vision (CV) techniques, there has been a growing interest in plant disease recognition using leaf images (Qadri et al., 2024). Convolutional neural networks (CNNs) have become the most popular approach to image-based disease classification and have shown outstanding performance in benchmark datasets collected in controlled greenhouse settings (Balafas et al., 2023). The transfer learning strategies that have been developed using architectures pre-trained on large-scale natural image corpora, like ResNet (He et al., 2016), VGG (Simonyan and Zisserman, 2014), EfficientNet (Tan and Le, 2019), and DenseNet (Huang et al., 2017), have also been able to speed up progress by decreasing the amount of labelled data that is needed for a specific domain (Liu et al., 2021a). To boost discriminative disease-related features while selectively suppressing irrelevant background interference, attention mechanisms such as channel-wise squeeze-and-excitation (SE) blocks (Hu et al., 2018) and spatial attention modules have been added to CNN backbones (Zhang et al., 2024).

Although these developments have been made, the automated recognition of tomato leaf diseases in real-world field images is still a difficult image processing problem that existing deep learning methods fail to solve well. The main challenges are three important image processing problems: (i) high intra-class visual variation due to different stages of disease, lighting conditions, and cultivar differences; (ii) strong inter-class visual similarity, where lesions of different diseases, such as early blight (Yu et al., 2024) and target spot, or bacterial spot (Foolad et al., 2008) and septoria leaf spot (Abdulridha et al., 2020) exhibit overlapping color, texture, and morphological patterns that confuse standard convolutional filters; and (iii) severe image degradation under field conditions, including low contrast, motion blur, specular reflections from leaf surfaces, and complex cluttered backgrounds (Concepcion et al., 2020). These factors severely affect the performance of generic CNN architectures trained mainly on clean laboratory-acquired images like those provided in the PlantVillage (Mohanty et al., 2016) dataset. Moreover, the traditional pre-processing techniques are not suitable for recovering the fine-grained textural details and lesion boundary information required for early disease discrimination (Yang et al., 2022).

Although several models tested on this dataset achieved classification rates of over 95%, their accuracy was significantly reduced when they were applied to field-captured images for agricultural purposes. This indicates a significant difference between laboratory and field environments (Li et al., 2021). Single-dataset benchmarking has been revealed as a limitation in recent studies, and a need for using diverse image sources and domain-adaptive training techniques has been suggested (Ashurov et al., 2025). This mismatch between controlled laboratory images and real field images results in poor performance of the models when applied to real agricultural images, which necessitates special image processing challenges and solutions such as special preprocessing and architecture designs. The contributions of the study are as follows:

- This study presents the PlantCity (Khan et al., 2025) dataset, which contains real-field tomato leaf images collected from various regions of Pakistan, and merges it with some of the classes from the PlantVillage (Mohanty et al., 2016) dataset to form a more challenging benchmark of 14 classes, which closely resembles real-world agricultural conditions.
- A lightweight CNN architecture, called GSEBNet, is proposed in this study, which integrates Squeeze-and-Excitation (SE) blocks in Mobile Inverted Bottleneck Convolution (MBConv) layers with a custom depth-width progression and SE placement, along with a domain-specific GUBR preprocessing filter. The novelty is in the synergistic, task-specific integration of these well-known techniques, not in the introduction of new operators.
- The proposed framework achieves state-of-the-art performance on the 14-class dataset while maintaining a compact model size potentially suitable for resource-constrained devices given its compact size.

The rest of the work is organized as follows: Section 2 reviews related research on deep learning for tomato leaf disease classification. Section 3 details the dataset, preparation steps, and the proposed method. Section 4 presents experimental results and comparisons. Sections 5 and 6 conclude and discuss future research.

## 2 Related work

There are numerous applications of machine learning in various fields. The challenge of feature engineering, or the selection and transformation of input variables (features) to improve model learning, is a constant one. Deep neural networks have demonstrated significant success in the field of plant pathology, particularly in the automatic detection and extraction of relevant features from raw inputs, which alleviates the need for manual feature engineering. The work on tomato leaf disease (TLD) classification done so far is grouped by methodological theme, and the specific limitation that each theme does not address is emphasized before placing the proposed GSEBNet in the context of these limitations.

### 2.1 Transfer-learning and ensemble approaches

Some works use off-the-shelf, ImageNet-pretrained backbones in a transfer learning or ensembling fashion (Al-Gaashani et al., 2022). used feature concatenation and an ensemble of MobileNetV2 and NASNetMobile to achieve 97% accuracy on six PlantVillage classes (Javidan et al., 2024). Fused color, texture, and shape features with a weighted ensemble (95.585% accuracy), while (Alzahrani, 2023) fused DenseNet169, ResNet50V2, and a Vision Transformer (99% accuracy). Other works (Elfatimi, 2024) (Thangaraj et al., 2021), (Guerrero-Ibanez and Reyes-Muñoz, 2023), (Ulutaş and Aslantaş, 2023), used additional variants of transfer learning on the same PlantVillage benchmark. All of these methods are tested only on the curated, laboratory-captured PlantVillage dataset and do not adapt the network to the fine-grained, low-contrast nature of leaf disease images, except for the high accuracy achieved by these approaches.

### 2.2 Attention-augmented and residual CNN architectures

To enhance discriminative feature learning (Zhao et al., 2021), introduced attention modules to a residual CNN 96.81% accuracy, while (Zhou et al., 2021) proposed a restructured residual dense network, achieving 95% top-1 accuracy (Bhujel et al., 2022). added attention modules to a lightweight CNN, achieving 99.69% accuracy on 11 classes, and (Peng et al., 2023) proposed DIMPCNET, which is a CNN with an attention module on a self-collected five-class dataset (94.445% accuracy). In each of these works, the attention block is added to a pre-existing backbone, not explicitly designed with an image-enhancement stage for degraded field imagery, and none test beyond PlantVillage or small, single-source collections.

### 2.3 Lightweight and mobile-oriented architectures

Several studies seek parameter efficiency in the context of deployment constraints in agricultural settings (Islam et al., 2022). compared activation functions in a compact CNN (97.5% validation accuracy); (Chen et al., 2022) adapted AlexNet for a mobile app (98% accuracy); (Ahmed et al., 2022) and (Rashid et al., 2025) modified MobileNetV2/V3 with inverted residual blocks; (Alnamoly et al., 2024) used depthwise-separable convolutions to reach 99.10% accuracy with only 221,594 parameters; and (Peyal et al., 2023) built a 9.5-million-parameter 2D CNN for real-time Android inference of 4.84 ms/image (Paul et al., 2023). and (Yildiz et al., 2024) developed lightweight VGG-based mobile applications. All of these methods focus on inference speed and model size, but are trained and tested on PlantVillage or small single-source collections, and none have a dedicated preprocessing stage to account for the illumination and contrast variation of field-captured images. To classify plant diseases on resource-constrained IoT devices, a recent study (Radojcic et al., 2025) introduced a two-layer hybrid architecture, where the first layer consists of CNNs and the second layer is boosting models (AdaBoost/CatBoost), optimized by a modified particle swarm algorithm, which enables efficient on-device inference for Agriculture 4.0 applications.

### 2.4 Hybrid, fusion, and alternative deep learning architectures

Another line of work is architectural hybridization (Zhang et al., 2022). integrated multimodal data and transfer learning on ResNet34 with 98.9% accuracy; (Sanida et al., 2023) proposed hybrid CNNs with an accuracy of 99.17–99.23%; (Thangaraj et al., 2024) used multi-level feature fusion within a modified Xception model with 99.61% accuracy; (Khan et al., 2024) fused deep and Bayesian-optimized machine learning models achieved 98.52% accuracy; (Abouelmagd et al., 2024) proposed an optimized CapsNet with 96.39% accuracy; (Irmak and Saygili, 2024) fused handcrafted feature extractors HOG, LBP, GLCM with classical classifiers KNN, ELM, SVM; and (Assaduzzaman et al., 2025) proposed a hybrid model of multi-scale fusion with squeeze-and-excitation blocks on EfficientNetB0 with 99.11% accuracy. These hybrid designs offer greater accuracy by combining architectural or feature-level fusion, but nearly all still rely on PlantVillage, whose imaging conditions are controlled in the lab, which is quite different from the lighting and background conditions found in the field.

### 2.5 Vision transformers and modern CNN backbones

In recent years, the focus in visual representation learning has turned to hierarchical Vision Transformers (ViTs) and their convolutional counterparts. Swin-Transformer (Liu et al., 2021b) proposes shifted-window self-attention for efficient multi-scale feature extraction, and ConvNeXt (Liu et al., 2022) updates the pure CNN architecture with large-kernel depthwise convolutions and layer normalization, thereby matching ViT performance. While effective on natural-image benchmarks, these general-purpose backbones are largely under-explored for fine-grained agricultural disease recognition, where subtle texture and lesion-boundary cues are important, and computational budgets are limited.

### 2.6 Research gap and positioning of the proposed work

The studies reviewed above and their datasets, class counts, and methods are summarized in **Table 1**. There are three uniform gaps in the categories listed in Sections A-E. First, the vast majority of methods are tested only on PlantVillage, a curated dataset collected in the lab that does not capture the lighting variability, background noise, and image degradation found in field-acquired images. Second, general-purpose backbones (MobileNet, MobileNetV2 (Sandler et al., 2018), DenseNet, VGG, ResNet, and the modern Swin-Transformer/ConvNeXt families) are used extensively without adaptation to the specific challenges of TLD imagery: high intra-class similarity of disease symptoms, subtle inter-class variation, and inconsistent illumination. Thirdly, if attention mechanisms are employed, they are not co-designed with an image-enhancement stage, and the two stages do not work synergistically.

**Table 1:** Summary of the related work.

Reference	Year	Dataset	Classes	Method	Key limitation
(Al-Gaashani et al., 2022)	2021	PlantVillage	6	Transfer Learning-Based Ensemble Model (MobileNetV2 and NASNetMobile)	PlantVillage only; generic ensemble, no architectural adaptation
(Zhao et al., 2021)	2021	PlantVillage	10	Attention mechanism-based CNN	PlantVillage only; attention bolted onto generic backbone
(Zhou et al., 2021)	2021	PlantVillage	9	Deep Residual Dense Network (RDN)	PlantVillage only; added complexity, no field-image handling
(Islam et al., 2022)	2022	PlantVillage and Plant Factory, Ehime University	7	Use different activation functions and Batch normalization on CNN.	PlantVillage-based; no field preprocessing
(Bhujel et al., 2022)	2022	PlantVillage and 1 class from South Korea.	11	Lightweight Attention-Based Network Design	PlantVillage + 1 class; attention not co-designed with preprocessing
(Zhang et al., 2022)	2022	PlantVillage and Own Collected	7	Multimodal Data Fusion Recognition Multi-ResNet34 Model.	Small multimodal dataset; unmodified ResNet34
(Chen et al., 2022)	2022	PlantVillage	10	Modified AlexNet	PlantVillage only; generic AlexNet backbone
(Ahmed et al., 2022)	2022	PlantVillage	10	TL-based and modified MobileNetV2	PlantVillage only; generic MobileNetV2
(Sanida et al., 2023)	2023	PlantVillage	10	Efficient hybrid CNN	PlantVillage only; no field-image preprocessing
(Javidan et al., 2024)	2023	PlantVillage	7	Ensemble model	PlantVillage only; no field validation
(Alzahrani, 2023)	2023	PlantVillage	10	Many TL-based models	PlantVillage only; general-purpose backbones
(Peng et al., 2023)	2023	Own dataset collection	5	Proposed model DIMPCNET	Small 5-class self-collected data; no public benchmark
(Thangaraj et al., 2024)	2023	PlantVillage	10	Modified Xception based on Multi-Level Feature Fusion	PlantVillage only; unmodified Xception backbone
(Alnamoly et al., 2024)	2024	PlantVillage	10	Lightweight CNN	PlantVillage only; no preprocessing for field artifacts
(Khan et al., 2024)	2024	PlantVillage	10	Proposed Bayesian hybrid learning	PlantVillage only; ensemble raises inference cost
(Abouelmagd et al., 2024)	2024	PlantVillage	10	Capsule neural network (CapsNet	PlantVillage only; CapsNet, high computational cost
(Irmak and Saygili, 2024)	2024	PlantVillage	10	Different Feature extractors and machine learning classifiers	PlantVillage only; handcrafted features limit generalization
(Assaduzzaman et al., 2025)	2025	Tomato Disease: Multiple Sources	11	EfficientNetB0 with SE blocks and multi-scale feature fusion	Multi-source but no dedicated preprocessing stage
(Paul et al., 2023)	2025	PlantVillage and 1 own collection	11	TL based VGG16/19	Small 1-class own collection; standard VGG backbones
(Rashid et al., 2025)	2025	PlantVillage	10	Lightweight Modified MobileNetV3	PlantVillage only; no preprocessing stage
(Yildiz et al., 2024)	2025	PlantVillage	5	Utilized CNN models	PlantVillage only; standard CNN/ML comparison
(Liu et al., 2021b)	2021	ImageNet	—	Swin-Transformer	ImageNet only; unvalidated on disease imagery
(Liu et al., 2022)	2022	ImageNet	—	ConvNeXt	ImageNet only; unvalidated on disease imagery

To fill these gaps, the present work proposes a task-specific, lightweight architecture that (i) is trained and evaluated on a combined dataset of both PlantVillage and our field-collected PlantCity (Khan et al., 2025) dataset, instead of PlantVillage alone, (ii) strategically integrates MBConv blocks with Squeeze-and-Excitation modules using a custom depth-width progression and SE placement, rather than a generic backbone, and (iii) is preceded by a purpose-built Gamma-based Unsharp Brightness Reduction (GUBR) pre-processing stage that jointly addresses contrast, blur, and illumination artifacts. The contribution of GSEBNet is therefore the co-design and task-specific integration of these established components, evaluated under real field conditions, rather than the proposal of new individual operators.

## 3 Methodology

In the current research, a DL approach based on SE blocks and MBConv layers is presented for the classification of tomato leaf diseases. First, we have a collection of our own dataset, PlantCity (Khan et al., 2025), and expand the dataset with more classes to combine with the PlantVillage (Mohanty et al., 2016) dataset and split the combined dataset into 70% training, 10% validation, and 20% testing. **Figure 1** demonstrates the flowchart of the proposed model.

**Figure 1:**
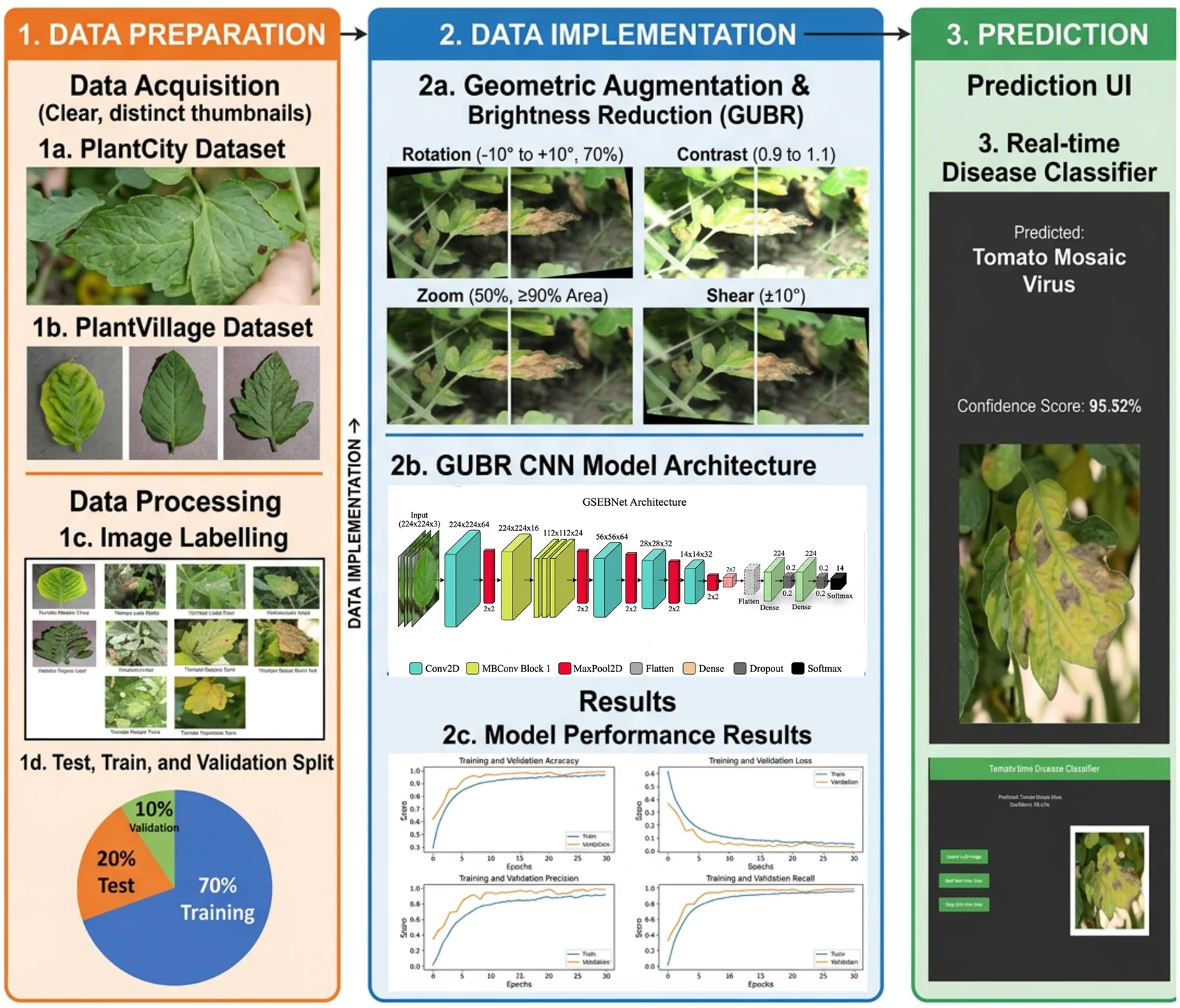
Flowchart of the proposed framework. **(1)** Data Preparation: **(1a)** PlantCity dataset, **(1b)** PlantVillage dataset, **(1c)** Image processing and labelling, **(1d)** Train/validation/test split. **(2)** Data Implementation: **(2a)** Geometric augmentation and GUBR brightness reduction, **(2b)** GSEBNet (GUBR CNN) model architecture, **(2c)** Model performance results. **(3)** Prediction: Real-time disease classifier interface.

### 3.1 Dataset description

Two datasets were utilized in this study: PlantCity (Khan et al., 2025), a proprietary collection, and PlantVillage (Mohanty et al., 2016), a publicly available resource. The dataset (Khan et al., 2025) is suitable for classification, testing, and training. It consists of 11 classes, one for healthy tomato leaves and 10 classes for various tomato leaf diseases, including bacterial spot, early blight, late blight, leaf miner, leaf curl, leaf mold, tomato spider mites, verticillium wilt, Fusarium wilt, and Septoria leaf spot. In addition, three classes from the PlantVillage dataset are added: tomato target spot, tomato yellow leaf curl virus, and tomato mosaic virus. All plant experiments complied with institutional policies and legal requirements. Sample images from the dataset are shown in **Figure 2**, and the number of images in each sample is summarized in **Table 2**. The images were taken during the field visits at various locations in Pakistan and multiple images were collected for each plant, which is considered in the data splitting process described in Section 3.2.

**Figure 2:**
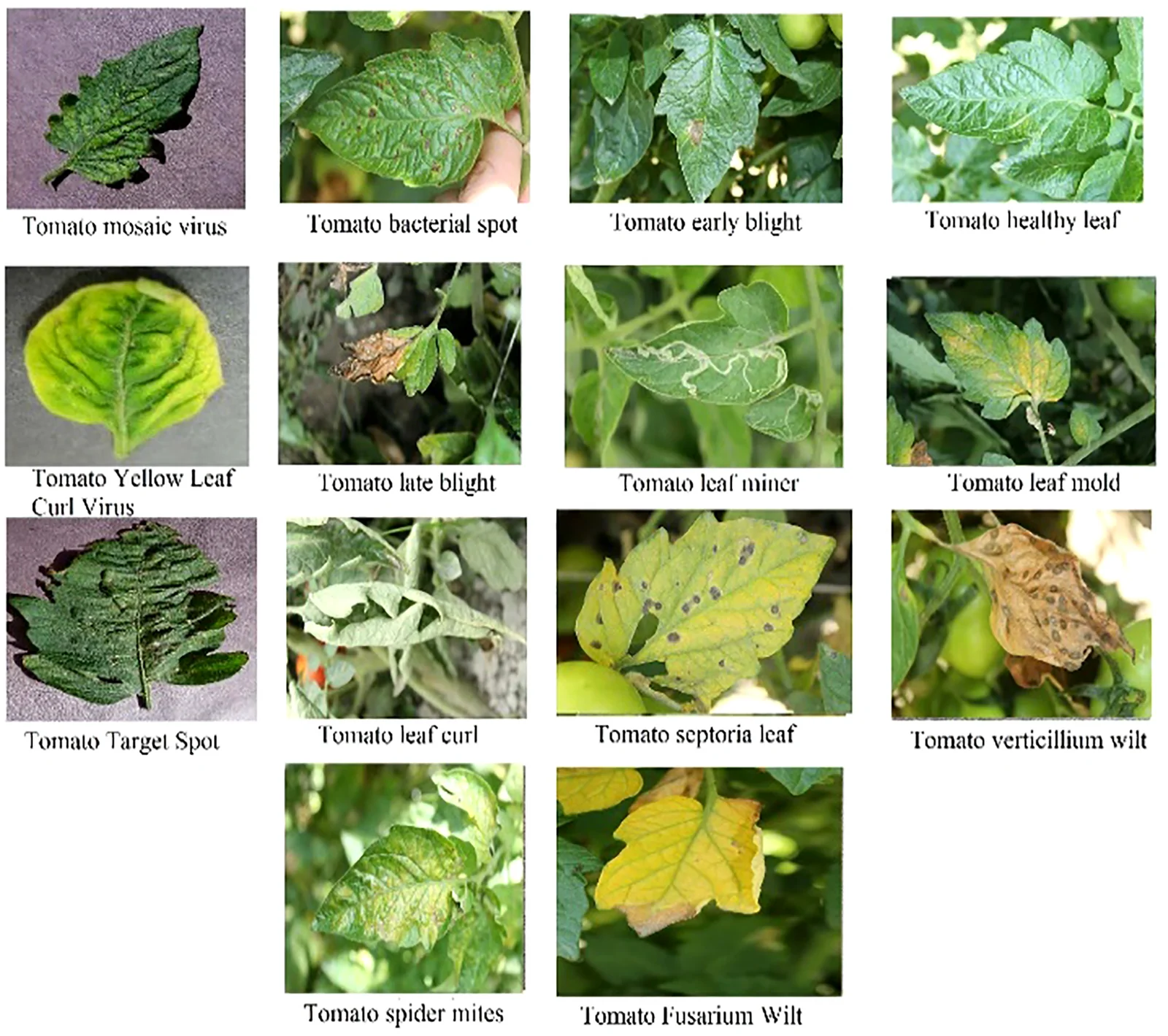
Sample images from the 14 classes of the combined dataset.

**Table 2:** Number of images in each class of the combined dataset.

Class name	Training	Validation	Test	Total
Tomato bacterial spot	1134	171	400	1705
Tomato early blight	1374	151	400	1925
Tomato healthy leaf	1330	148	400	1878
Tomato late blight	1011	112	301	1424
Tomato leaf miner	1349	148	400	1897
Tomato leaf curl	1332	148	413	1893
Tomato leaf mold	1359	151	400	1910
Tomato Septoria leaf	1132	126	347	1605
Tomato spider mites	457	51	127	635
Tomato verticillium wilt	378	42	105	525
Tomato Fusarium Wilt	281	31	89	401
Tomato mosaic virus	350	50	100	500
Tomato Target Spot	350	50	100	500
tomato_Yellow_Leaf_Curl_Virus	350	50	100	500
Total	12187	1429	3682	17298

The PlantCity subset was collected under real field conditions in April-July 2023 and 2024 in Charsadda (Village Sarki, 34.15°N, 71.74°E, 40-44 °C) and Chitral (Village Danin, 35.85°N, 71.79°E, 25-30 °C), Khyber Pakhtunkhwa, Pakistan. The images of tomato leaves were captured from 20–25 different field locations in Charsadda with different plants, disease stages, and natural light conditions (morning 7–10 AM and afternoon 2–5 PM). The images were taken with three different devices (Canon EOS 1200D (5184 × 3456), Infinix Hot 10 (4608 × 3456), and Infinix Hot 5 (1872 × 4160)) at a distance of 30–50 cm from the leaves with an angle of 45–90°, creating realistic variations in resolution, color response, and background noise. Disease identities were confirmed on site by consulting with field workers and agricultural specialists, who confirmed the disease type and symptoms before labelling, and were then annotated with the disease class during dataset construction, for each image. The full protocol for the acquisition, pre-processing, and annotation of PlantCity.

It is important to note that the four classes from PlantVillage (tomato spider mites, tomato target spot, tomato yellow leaf curl virus, and tomato mosaic virus) were collected under different imaging conditions than the ten PlantCity classes. This adds a potential source-confounding factor. We explicitly performed cross-domain experiments to quantify the impact of this domain difference.

### 3.2 Data preprocessing

Convolutional neural networks (CNNs) have fixed input image sizes. To satisfy these requirements, the images were first resized to 1200 × 800 and 800 × 1200 pixels and then resized to 224 × 224 pixels before training the proposed model. This pre-processing helped to standardize the input data, enabling the model to identify relevant information effectively.

The dataset was split into 70% training, 10% validation, and 20% testing, and data augmentation (random rotation, horizontal/vertical flip, shear, and zoom) was applied only to the training set to improve generalization. No additional oversampling of minority classes or class-weighting was performed; the original class distribution was retained. To guarantee an independent and unbiased evaluation, only original images were used for the validation and test sets. Random rotations (between −10° and +10° with a 70% probability), contrast adjustment (between 0.7 and 1.3), random zoom (between 50% and 90% with a 70% probability), and shearing (± 10°). Examples of the applied data augmentation techniques are shown in **Figure 3**. The augmentation is applied specifically to reduce the model’s reliance on dataset-specific background/camera characteristics, and the validation/test sets use only original images so that generalization is measured on unmodified data.

**Figure 3:**
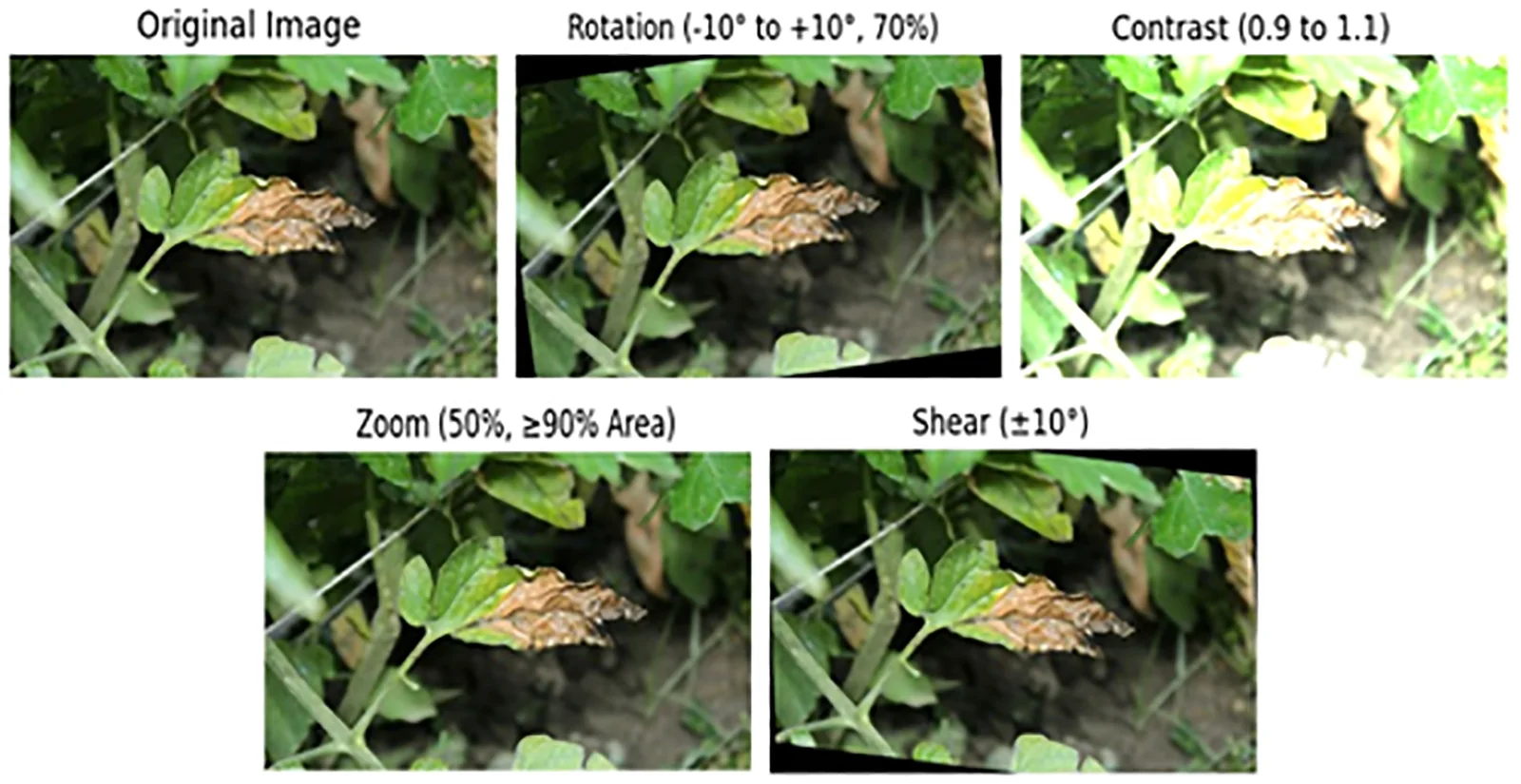
A sequence of an image when applying data augmentation.

The split was done at the image level, and stratified by class to maintain the same class distribution across the training, validation, and test sets. The partition was reproducible by using a fixed random seed. The division is strictly at the image level, but near-duplicate frames were visually identified and removed before splitting, not at the plant level or acquisition-session level. Therefore, it is not possible to completely exclude the possibility of a residual risk of highly similar images of the same plant being present in different subsets. In future extensions of the PlantCity benchmark, a more stringent plant-level splitting protocol will be used.

### 3.3 GUBR filter (gamma-unsharp brightness reduction filter)

A special pipeline for image enhancement that contains a linear brightness-reduction stage for brightness control, an unsharp masking stage for detail enhancement, and gamma correction for contrast enhancement. Image quality and feature differentiation are enhanced by this composite filter before model input. The GUBR filter is not a new image-processing operator, but a task-oriented sequence of classical gamma correction, unsharp masking, and linear brightness reduction, parameterized for the typical degradations of tomato leaf images captured in a field.

A composite Gamma-based Unsharp Brightness Reduction (GUBR) filter is suggested, which is specially designed for tomato leaf disease images captured in a real-field environment. It is based on classical techniques, but the sequential combination and parameter tuning in GUBR are effective in solving common problems such as low contrast, blur, and uneven lighting that are encountered in both PlantVillage and PlantCity (Khan et al., 2025) datasets.

All experiments were performed with the final set of parameter values obtained from a small grid search on the validation set. Both the PlantCity and PlantVillage images were processed with the same fixed GUBR parameters. While PlantVillage is a controlled lab dataset, many of the images in the dataset have low contrast, mild blur, or uneven illumination. The same pre-processing pipeline was found to be useful for both sources. The stable performance gains seen with the 11-class subset and the 14-class dataset (**Tables 3**, **4**) continue to validate the usefulness of these settings in both imaging conditions.

**Table 3:** Performance comparison of the proposed GSEBNet and baseline models on the PlantCity subset only (11 classes).

Methods	GBUR	Precision	Recall	Accuracy	Cohen’s Kappa
DenseNet121	×	98.33%	97.34%	97.99%	0.9775
DenseNet121	✓	98.71%	97.79%	98.21%	0.9801
DeseNet169	×	98.75%	97.99%	98.40%	0.9821
DeseNet169	✓	98.99%	98.26%	98.67%	0.9866
DenseNet201	×	98.11%	93.52%	97.25%	0.9692
DenseNet201	✓	98.38%	94.82%	97.69%	0.9714
VGG16	×	98.05%	95.35%	97.13%	0.9624
VGG16	✓	98.17%	95.77%	97.53%	0.9656
VGG19	×	97.55%	93.13%	96.67%	0.9537
VGG19	✓	97.82%	93.91%	96.91%	0.9579
MobileNet	×	99.11%	95.71%	98.58%	0.9841
MobileNet	✓	99.19%	96.41%	98.88%	0.9861
MobileNet V2	×	98.10%	95.18%	97.91%	0.9808
MobileNet V2	✓	98.22%	95.63%	98.11%	0.9827
Baseline CNN	×	98.85%	98.85%	98.85%	0.9865
Baseline CNN	✓	99.00%	98.99%	99.00%	0.9896
MB-Net block 1	×	99.20%	99.20%	99.20%	0.9871
**MB-Net block 1**	✓	**99.49%**	**99.49%**	**99.50%**	**0.9944**
MB-Net block 2	×	98.96%	98.96%	98.97%	0.9884
MB-Net block 2	✓	99.16%	99.09%	99.15%	0.9905
MB-Net block 3	×	98.87%	98.82%	98.88%	0.9872
MB-Net block 3	×	98.95%	98.96%	99.00%	0.9893

Bold values indicate the best performance results achieved by the proposed GSEBNet model.

**Table 4:** Performance comparison of the proposed GSEBNet and baseline models on the combined 14-class dataset (PlantCity + 3 PlantVillage classes).

Methods	GBUR	Precision	Recall	Accuracy	Cohen’s Kappa
DenseNet121	×	93.56%	97.14%	94.68%	0.9366
DenseNet121	✓	94.56%	97.56%	95.61%	0.9437
DeseNet169	×	98.08%	96.32%	97.32%	0.9705
DeseNet169	✓	98.24%	96.71%	97.66%	0.9762
DenseNet201	×	97.45%	96.04%	96.67%	0.9624
DenseNet201	✓	97.84%	96.63%	96.91%	0.9672
VGG16	×	94.46%	95.53%	95.91%	0.9578
VGG16	✓	98.62%	97.71%	98.17%	0.9791
VGG19	×	93.14%	86.99%	89.75%	0.8843
VGG19	✓	93.22%	94.61%	94.02%	0.9376
MobileNet	×	98.86%	97.81%	98.43%	0.9822
MobileNet	✓	99.00%	98.03%	98.69%	0.9855
MobileNet V2	×	98.02%	98.12%	97.98%	0.9765
MobileNet V2	✓	98.23%	98.43%	98.15%	0.9792
Baseline CNN	×	98.52%	98.62%	98.48%	0.9823
Baseline CNN	✓	98.63%	98.70%	98.56%	0.9829
MB-Net block 1	×	99.01%	98.79%	98.96%	0.9886
**MB-Net block 1**	✓	**99.48%**	**99.45%**	**99.48%**	**0.9943**
MB-Net block 2	×	98.58%	98.66%	98.53%	0.9827
MB-Net block 2	✓	98.70%	98.76%	98.60%	0.9866
MB-Net block 3	×	98.45%	98.60%	98.45%	0.9820
MB-Net block 3	×	98.54%	98.73%	98.52%	0.9801

Bold values indicate the best performance results achieved by the proposed GSEBNet model.

- Gamma correction: γ = 0.8
- Unsharp masking: sharpening coefficient λ = 1.5, Gaussian standard deviation σ = 1.0, kernel size = 5 × 5
- Linear brightness reduction: β = 0.9

The exact processing order applied to every image is:

1. Gamma correction
2. Unsharp masking
3. Linear brightness reduction

After each stage, pixel values are clipped to the valid range [0, 255], and the final output is converted back to 8-bit unsigned integer format before being fed to the network. The same fixed parameter set was used for all images in both the PlantCity and PlantVillage subsets.

Gamma correction is a non-linear modification that adjusts an image’s brightness. Enhancing or reducing specific intensity ranges is extremely helpful for fixing lighting in images. I(x, y) represents the input image, where pixel intensities range from [0, 255], and x and y denote spatial coordinates. The mathematical formulations of the gamma correction, inverse gamma, unsharp masking, and linear brightness reduction steps are given in Equations 1–4.

(1) $${\text{I}}^{\prime }(\text{x},\text{y})=255\times (\frac{\text{I}(\text{x},\text{y})}{255})\gamma$$

The inverse gamma is defined in the following way, and a lookup table (LUT) is employed to carry out this transformation:

(2) $${\text{γ}}^{-1}=\frac{1}{\text{γ}}$$

Unsharp masking emphasizes edges, enhancing intricate details in images. It entails blending the original image with a scaled version after subtracting a blurred version of the image. The unsharp masking operation can be expressed mathematically as follows:

(3) $${\text{I}}_{\text{sharp}}(\text{x},\text{y})={\text{I}}_{\text{gamma}}(\text{x},\text{y})+{\text{λ[I}}_{\text{gamma}}{\left(\text{x},\text{y}\right)}_{\;-\;\;}\text{G}({\text{I}}_{\text{gamma }}(\text{x},\text{y}))]$$

Where:

-
$I_{gamma}(x,y)=image\;after\;gamma\;correction$
-
G(.)=Gaussion blur operator 
-
λ=sharping streght parameter 

The last step involves performing a linear brightness adjustment. The meaning of this direct pixel-wise scaling is:

(4) $${\text{I}}_{\text{final}}(\text{x},\text{y})=\text{δ}\times {\text{I}}_{\text{sharp}}(\text{x},\text{y})+\text{β}$$

Note that the GUBR filter is not suggested as a new image processing operator. Instead, it is based on task-specific engineering, which involves the sequential application of gamma correction, unsharp masking, and linear brightness reduction to the imagery of tomato leaf disease. Plant disease datasets often have uneven illumination, outdoor shot motion blur, and low contrast between diseased and healthy tissue areas. The GUBR pipeline addresses these challenges in a computationally efficient way and is suitable for implementation on resource-constrained edge devices in precision agriculture applications.

**Figure 4** shows the applied GUBR filter on the image to enhance the low-quality images, especially the PlantVillage dataset, which has low-quality images, and many pictures are blurred and dark.

**Figure 4:**
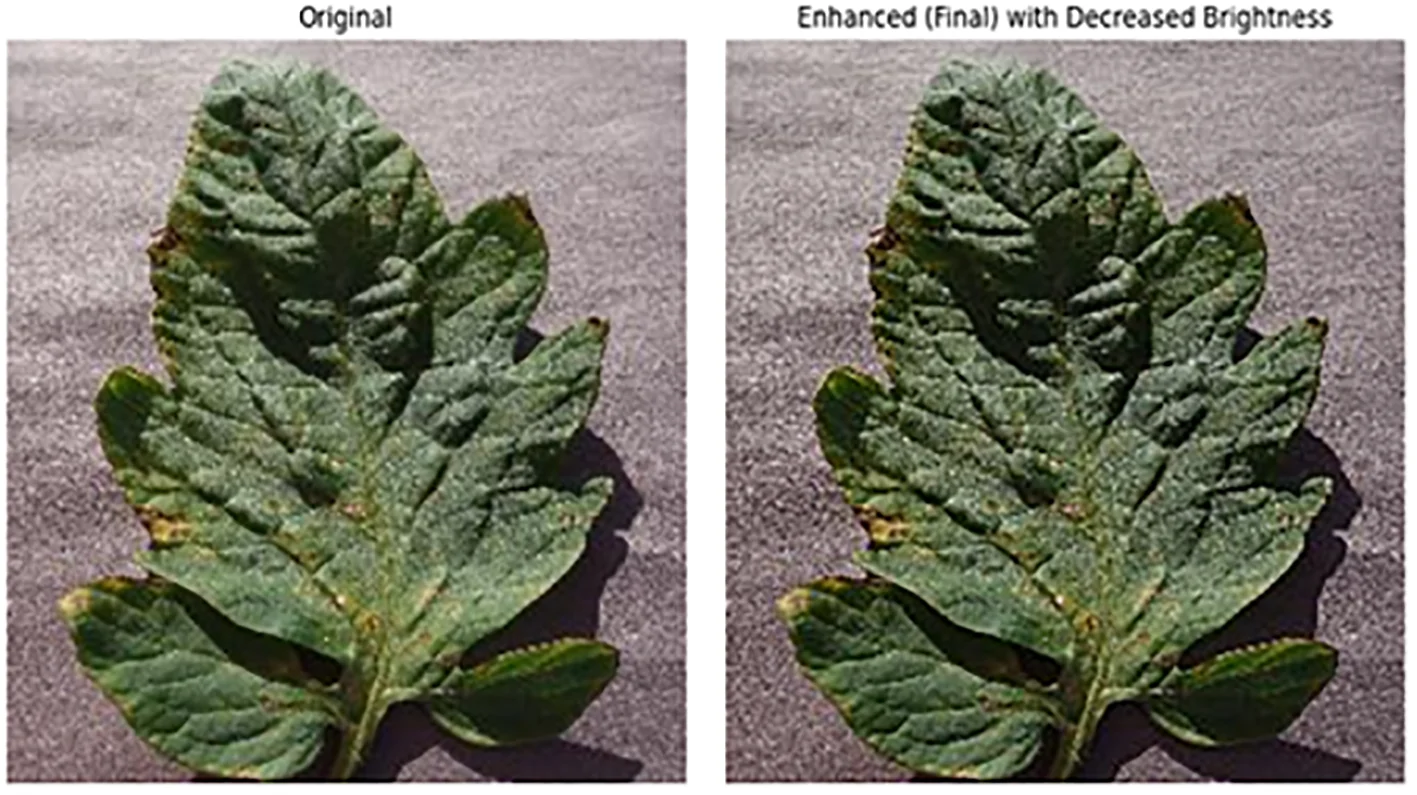
The difference between the original and the GUBR filter-applied image.

### 3.4 Model architecture

The convolutional layer’s reduced filter size extracts low-level features from input images, providing a foundation for further processing. Robust feature generalization enables improved learning, even with diverse images. We use multiple GPUs to reduce computation time and group convolutional layers to split processing, increasing training effectiveness. Our GSEBNet model stands out by integrating Mobile Inverted Residual Bottleneck (MBConv) blocks with Squeeze-and-Excitation (SE) modules, which together enhance feature extraction and computational efficiency. **Figure 5** shows the GSEBNet model.

**Figure 5:**
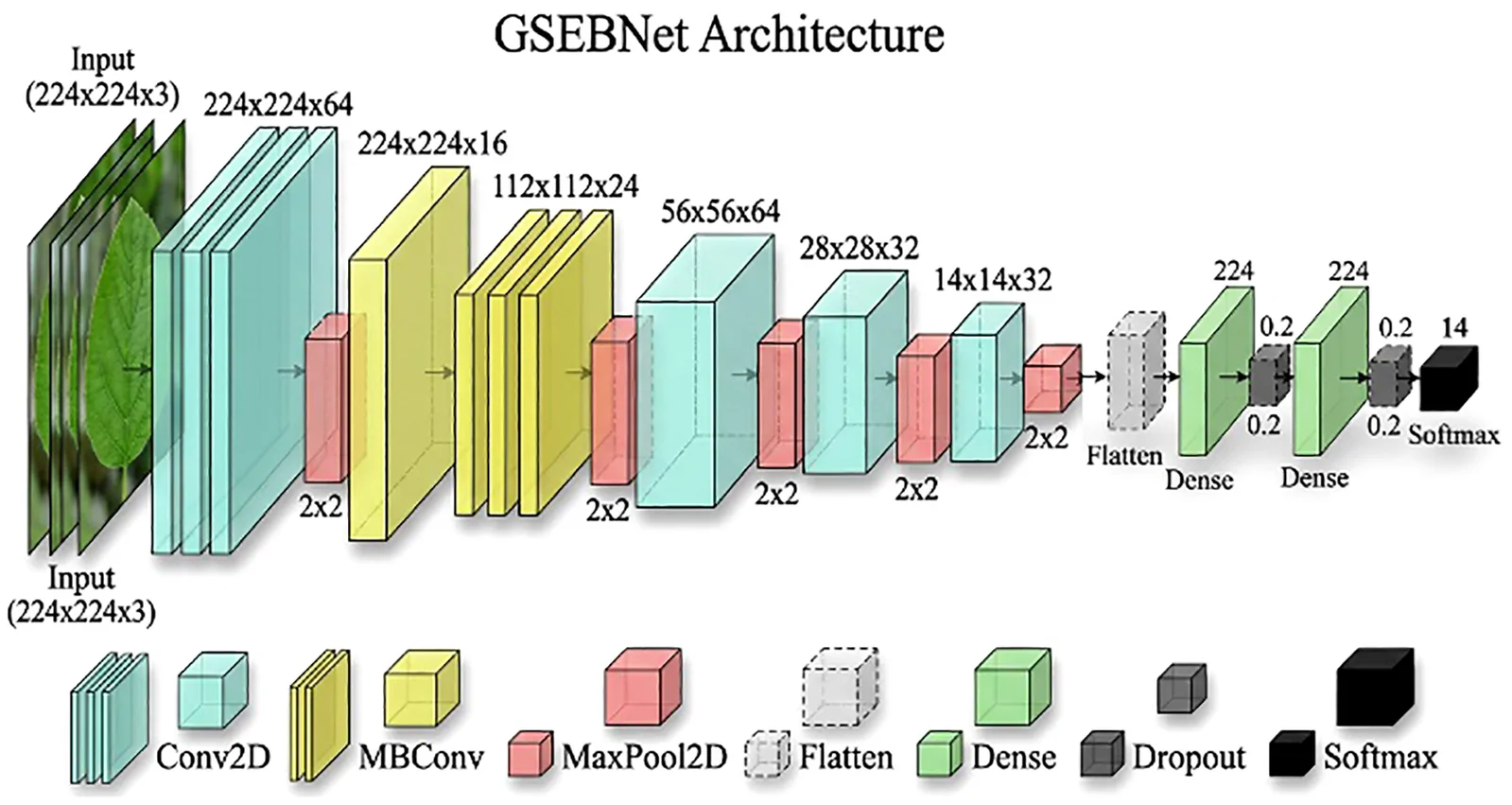
The proposed GSEBNet model.

The proposed GSEBNet is different from other related works, such as EfficientNetB0+SE (Assaduzzaman et al., 2025) and modified MobileNetV3 (Rashid et al., 2025), in two ways: (1) SE blocks are intentionally placed within MBConv layers using different expansion ratios, and (2) the GUBR preprocessing stage is inserted before the CNN backbone. The motivation behind these design decisions is to better capture subtle disease textures and to better focus on channel-wise attention under real-world field conditions. The novelty of GSEBNet is the custom-made embedding of SE blocks at specific locations in the MBConv structure, as well as the pre-processing stage of GUBR. It is not just a simple stacking, but a well-designed pipeline that greatly improves feature recalibration for poor-quality field images. The depth-width progression for SE blocks is different from EfficientNetB0+SE and the modified MobileNetV3, which follow a standard compound scaling or inverted-residual schedule, respectively. The depth-width progression for SE blocks is custom, with SE blocks deliberately added after each phase of expansion in MBConv. Furthermore, the expansion ratios and the number of MBConv stages are specially selected for the fine-grained texture of tomato leaf diseases. The architecture also distinguishes itself from EfficientNetB0+SE and the modified MobileNetV3 designs by the previous GUBR preprocessing stage.

The model consists of several convolutional layers, MBConv blocks, max-pooling layers, and fully connected layers that process input images with dimensions of 224 × 224 × 3. **Table 5** summarizes the architecture.

**Table 5:** The internal architecture of the proposed GSEBNet model.

Layer	Parameters
Input	224 × 224 × 3
Conv2D	64 filters, 3×3, Swish activation
MaxPooling2D	2×2
MBConv Block 1	16 filters, 3×3, SE ratio 0.25, stride 1, expand 1×
MBConv Block 2	24 filters, 3×3, SE ratio 0.25, stride 2, expand 6×
MaxPooling2D	2×2
Conv2D	64 filters, 3×3
MaxPooling2D	2x2
Conv2D	32 filters, 3×3
MaxPooling2D	2x2
Conv2D	32 filters, 3×3
MaxPooling2D	2x2
Flatten	-----
Dense	224 units, ReLU
Dropout	20%
Dense	224 units, ReLU
Dropout	20%
Dense	14 units, Softmax

Along with the MBConv block, EfficientNet also applies the SE block, resulting in the structure shown in **Figure 6**.

**Figure 6:**
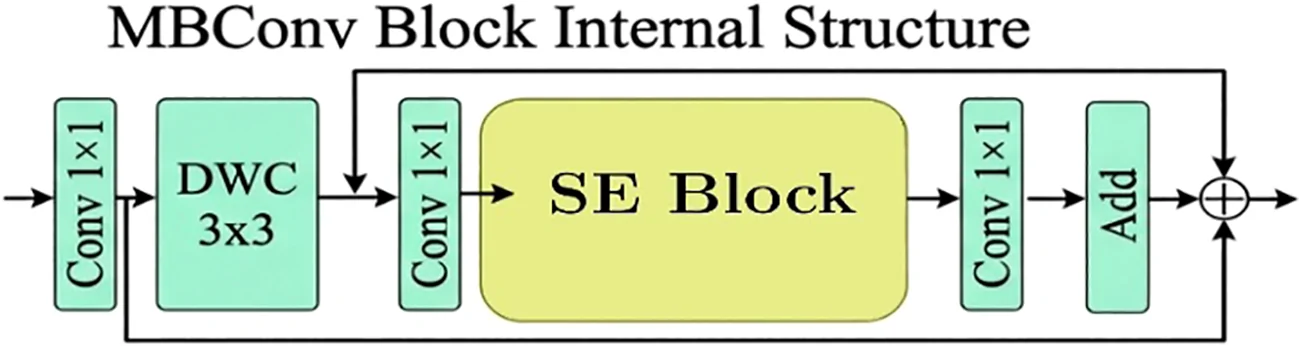
Showcase of MBConv block layers.

The specific placement of SE blocks after every MBConv expansion phase and the previous GUBR filter leads to a synergistic effect that enhances the attention of features in the channel-wise direction in the real-field condition for disease-specific patterns.

An MBConv block is applied with a stride of 1, a 3×33 kernel, 16 filters, and an expansion ratio of 1.

(5) $${\text{X}}_{1}=\text{MBConvBlock}({\text{X}}_{\text{in}}\;,\;1,16,3,1,0.25)$$

Where

$X{\_}_{in}$ is the input feature map.

${\text{X }}_{1}=\text{MBConvBlock}({\text{X}}_{\text{input}},\text{ expand}=1,\text{ filters}=16,\text{k}=3,\text{s}=1,{\text{se}}_{\text{ratio}}=0.25)$Following the first block, the second MBConv block is applied with a stride of 2, a 3x33 kernel, 24 filters, and an expansion ratio of 6. By using a stride of two, the spatial dimensions are downsampled.

(6) $${\text{X}}_{2\;}=MBConvBlock({\text{X}}^{1}\;,\;6,\;24,\;3,2,0.25)$$

Or simply:


$$X_{2\;}=MBConvBlock(x_{1}\;,expand=6,filters=24,k=3,s=2,\;se_{ratio}=0.25)$$

After the MBConv blocks, the spatial dimensions are gradually reduced by a sequence of max-pooling processes, and the features are further refined by additional convolutions.

(7) $${\text{X}}_{3}=\text{MaxPool}2{\text{D }}_{2\times \;2}({\text{X }}_{2})$$

The first two MBConv blocks and the subsequent max-pooling operation are formally defined in Equations 5–7. Following the convolutional processes, the SE block explicitly models the interdependencies across channels to recalibrate the channel-wise feature responses. As a result, the network will dynamically assign a higher weight to the most significant channels rather than assigning an equal weight to each channel. **Figure 7** below shows this block’s constituent parts. Moreover, **Figure 8** represents a visualization of the features of every layer from the proposed model.

**Figure 7:**
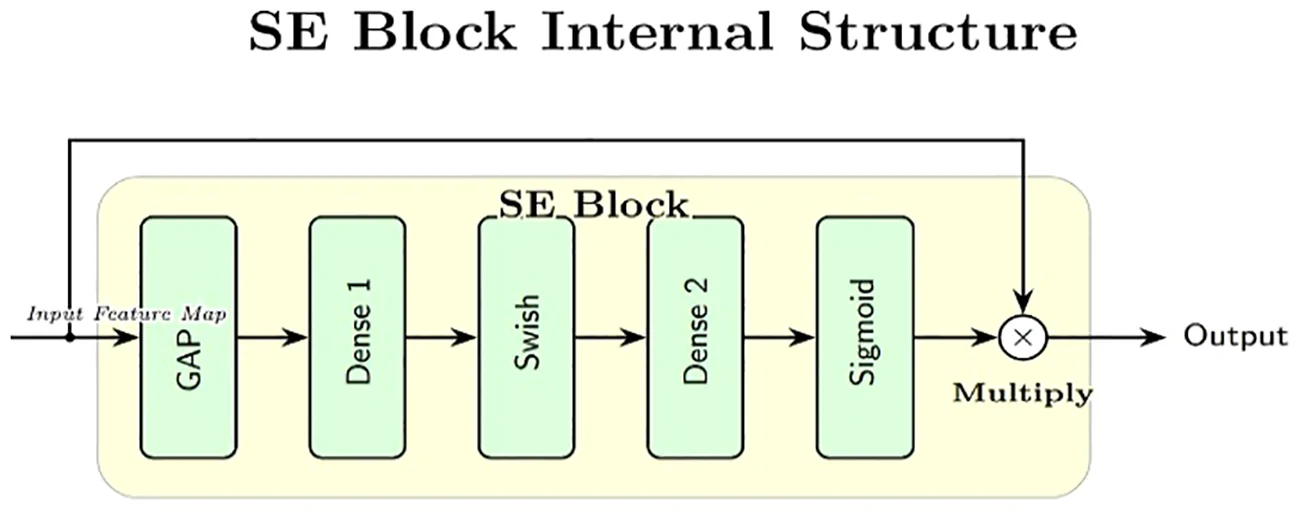
Detailed structure of squeeze-and-excitation (SE) block.

**Figure 8:**
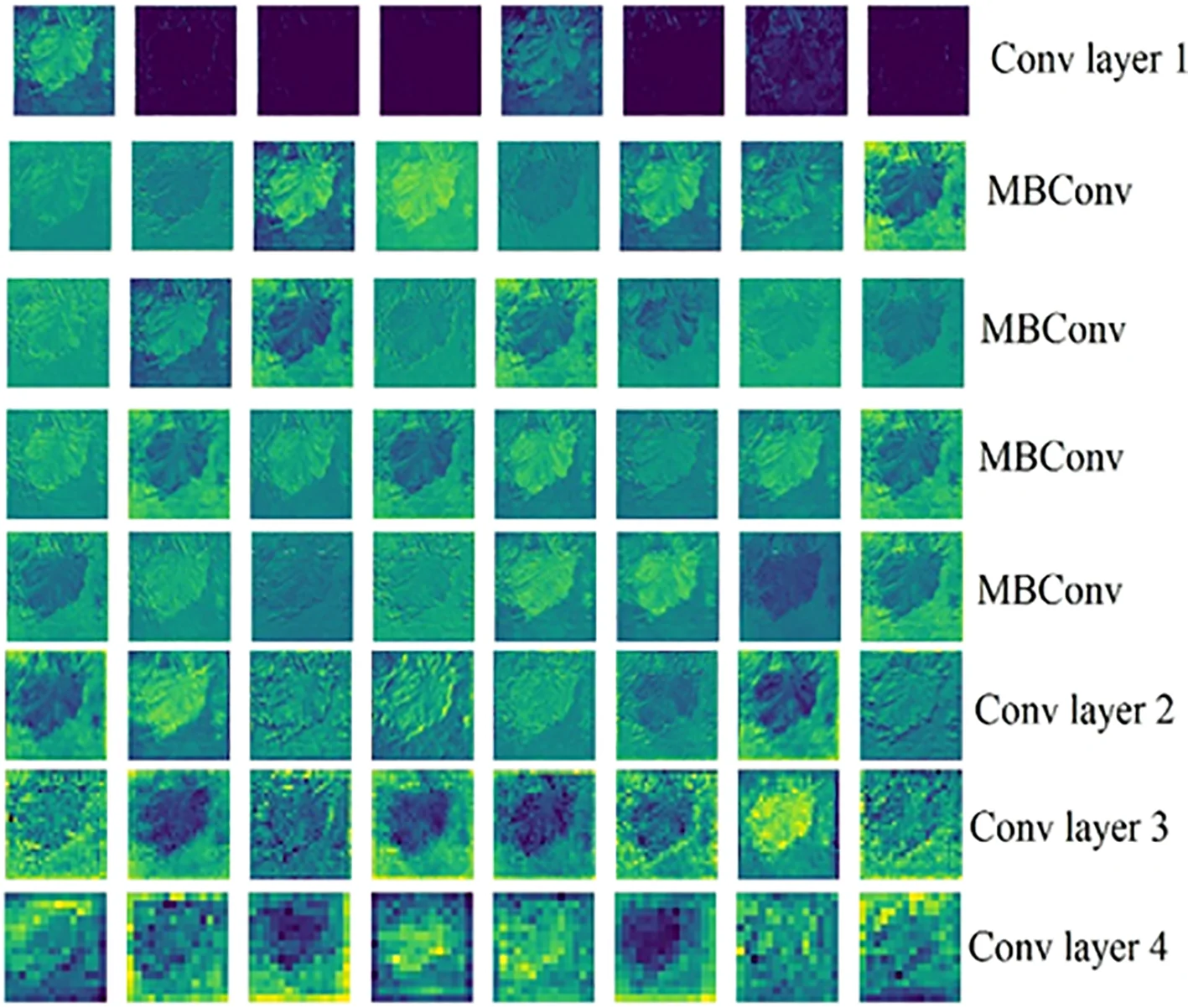
Some sample features of every layer of the proposed model.

The novelty of GSEBNet is the custom-made embedding of SE blocks at specific locations in the MBConv structure, as well as the pre-processing stage of GUBR. It is not just a simple stacking, but a well-designed pipeline that greatly improves feature recalibration for poor-quality field images.

### 3.5 Hyperparameter tuning

Based on preliminary testing, we manually adjusted hyperparameters, including learning rate, batch size, and dropout rate. Based on model convergence speed and validation performance, the final values (learning rate = 0.001, batch size = 26, dropout = 0.20) were selected. The entire set of parameters against every experiment is presented in **Table 6**.

**Table 6:** Summary of all hyperparameters used in the experiment.

Parameters for the classification model	Description
Image Size	224x224
convolution layers	Four
max-pooling layers	Four
Activation function	ReLU, Swish
The learning rate	0.001
Optimizer	Adam
Epochs	30
Batch size	26
Dropout Rate	0.20
Stride	1x1, 2x2
Image zooming	0.1
Shear range	0.1
Vertical flip	True
Horizontal flip	True
Gamma correction	γ = 0.6
Unsharp masking	σ = 1.5
sharpening weight	1.5
Brightness reduction	β = 0.9
Processing order	1. Gamma → 2. Unsharp → 3. Brightness
Final normalization	Scaled to [0, 1]

### 3.6 Experimental setup

To verify our techniques on new data and prevent overfitting, we undertake trials across various train-test splits. We use 70% of the dataset for training, 10% for validation, and 20% for testing. During training, we compute mean precision, recall, and total accuracy at regular intervals (at the conclusion of each epoch).

This experiment uses a built-in GPU-based setup with Windows 10 as the operating system, an Intel i7 3770 3.50 GHz CPU, and an RTX 2060 Super GPU with 20 GB of RAM and a 512 GB solid-state drive. Python 3.9.4 and the TensorFlow GPU 2.10 artificial neural network library are used as the training environment. The deep neural network acceleration package CUDA 11.2 is utilized concurrently.

### 3.7 The evaluation index

The classification report shows each class’s precision, recall, F1-score, accuracy, and support as performance parameters. On test data, it measures how well the trained models perform. Equation 8 expresses accuracy as the number of correct predictions divided by the total number of guesses from the confusion matrix.

(8) $$\text{Acuracy}=\frac{\text{TP}+\text{TN}}{\text{TP}+\text{TN}+\text{FP}+\text{FN}}$$

Precision calculates the proportion of predicted positives that are actually positive. It can be computed per class by applying Equation 9.

(9) $$\text{Pecision}=\frac{\text{TP}}{\text{TP}+\text{FP}}$$

Recall, which may be calculated per class using Equation 10, measures the number of accurately predicted positive records.

(10) $$\text{Recall}=\frac{\text{TP}}{\text{TP}+\text{FN}}$$

Equation 11 can be used to calculate the F1-score, which is the harmonic mean of precision and recall, for each class.

(11) $$\text{F}1\text{ Score}=\frac{2\times \text{Precision}\times \text{Recall}}{\text{Precision}+\text{Recall}}$$

When assessing how well human or ML annotators perform in tasks like classification or labeling, where multiple raters or annotators are involved, Cohen’s kappa, a statistical measure that accounts for agreement that occurs beyond chance agreement, is frequently employed. The formula for Cohen’s kappa is given in Equation 12.

(12) $$\text{k}=\frac{{\text{p}}_{\text{o}}-{\text{p}}_{\text{e}}}{1-{\text{p}}_{\text{e}}}$$

## 4 Results

### 4.1 Experiments

We have conducted several experiments for tomato leaf disease image classification schemes, as shown in **Tables 3**, **4**. Our proposed model outperforms multi-class problems. The accuracy obtained with our suggested model shows a substantial performance improvement, especially when considering the increase in class complexity. Our model’s resilience and versatility are demonstrated by the remarkable accuracy of 99.48% in 14 classes, and with 11 classes, we achieved a test accuracy of 99.50%. Our model’s superiority is demonstrated when compared to the benchmarks of MobileNet, MobileNetV2, VGG16, VGG19, DenseNet121, DenseNet169, and DenseNet201. These results, which are presented in **Tables 3**, **4** together with other metrics including recall, precision, accuracy, and Cohen’s Kappa scores, highlight the thorough assessment and verification of our model’s effectiveness on a range of performance fronts. All baseline models (DenseNet121, DenseNet169, DenseNet201, MobileNet, MobileNetV2, VGG16, and VGG19) were trained and evaluated using the same experimental protocol as the proposed GSEBNet. In particular, the same data splits, data augmentation, input resolution (224×224), number of epochs, batch size, and model selection criterion (best validation accuracy) were employed. The baseline architectures were initialized with the ImageNet pre-trained weights and then fine-tuned on the target dataset. The proposed GSEBNet, however, was trained from scratch. If applicable, the effect of the GUBR preprocessing was also assessed on the baselines under the same conditions.

The results are reported separately for the PlantCity subset (**Tables 3**, **7** classes) and for the combined 14-class dataset (**Table 4**) to avoid over-interpretation of the performance on the mixed-source dataset. The cross domain transfer performance is reported separately in **Table 8**.

**Table 7:** Comparison of computational complexity and inference efficiency between the proposed GEBSNet and state-of-the-art CNN architectures.

Method	Params (M)	FLOPs (G)	Model size (MB)	Inference time (ms/image)	FPS
DenseNet121	7.05M	2.83G	82.0	106.38	9.4
DenseNet169	12.66M	3.36G	146	109.72	9.1
DenseNet201	18.34M	4.29G	211	115.17	8.7
MobileNet	3.24M	0.55G	37.3	64.70	15.5
MobileNetV2	2.27M	0.28G	26.6	71.71	13.9
VGG16	14.72M	15.35G	168	90.27	11.1
VGG19	20.03M	19.51G	229	89.79	11.1
GSEBNet	0.48M	1.15G	3.84	69.79	14.3

All inference times were obtained on the RTX 2060 Super GPU described in Section 3.6.

**Table 8:** 5-fold cross-validation summary (mean ± std) of the main GSEBNet model on the 14-class dataset.

Experiment	Accuracy	Precision	Recall	F1-score
Fold-1	99.37% ± 0.13%	99.23% ± 0.19%	99.05% ± 0.21%	0.9912 ± 0.0018
Fold-2	99.12% ± 0.18%	98.89% ± 0.27%	98.75% ± 0.27%	0.9880 ± 0.0026
Fold-3	98.12% ± 0.25%	98.03% ± 0.32%	97.38% ± 0.39%	0.9763 ± 0.0034
Fold-4	99.09% ± 0.19%	98.73% ± 0.29%	98.67% ± 0.30%	0.9867 ± 0.0029
Fold-5	98.84% ± 0.21%	98.42% ± 0.33%	98.64% ± 0.28%	0.9849 ± 0.0029

**Tables 3**, **4** show how well our proposed GSEBNet model, the MBConv model, and the SE block-based GUBR-enhanced model perform when it comes to identifying and classifying tomato leaf diseases in both the training and validation phases, with 11 and 14 classes, respectively. Remarkably, our model achieved an accuracy of 97.10% during training, demonstrating its strong learning ability. This was achieved in a training time of almost 7,200 seconds in the specified environment. Furthermore, our proposed model achieved a test accuracy of 99.48% on the held-out 14-class test set. In this research, the confusion matrix of the proposed GSEBNet model (**Figure 9**) was used to provide a detailed visualization of the model’s classification performance and to identify potential misclassifications between categories. The training and validation performance curves of the GSEBNet model for 14-class and 11-class configurations are shown in **Figure 10**, which demonstrate the convergence behavior, accuracy improvement, and generalization of the model during training. In addition, **Table 9** shows a detailed per-class analysis of the GSEBNet model in the 14-class scenario, with the precision, recall, and F1-score for each class, to assess the model’s performance and identify its strengths and weaknesses across the different classes.

**Figure 9:**
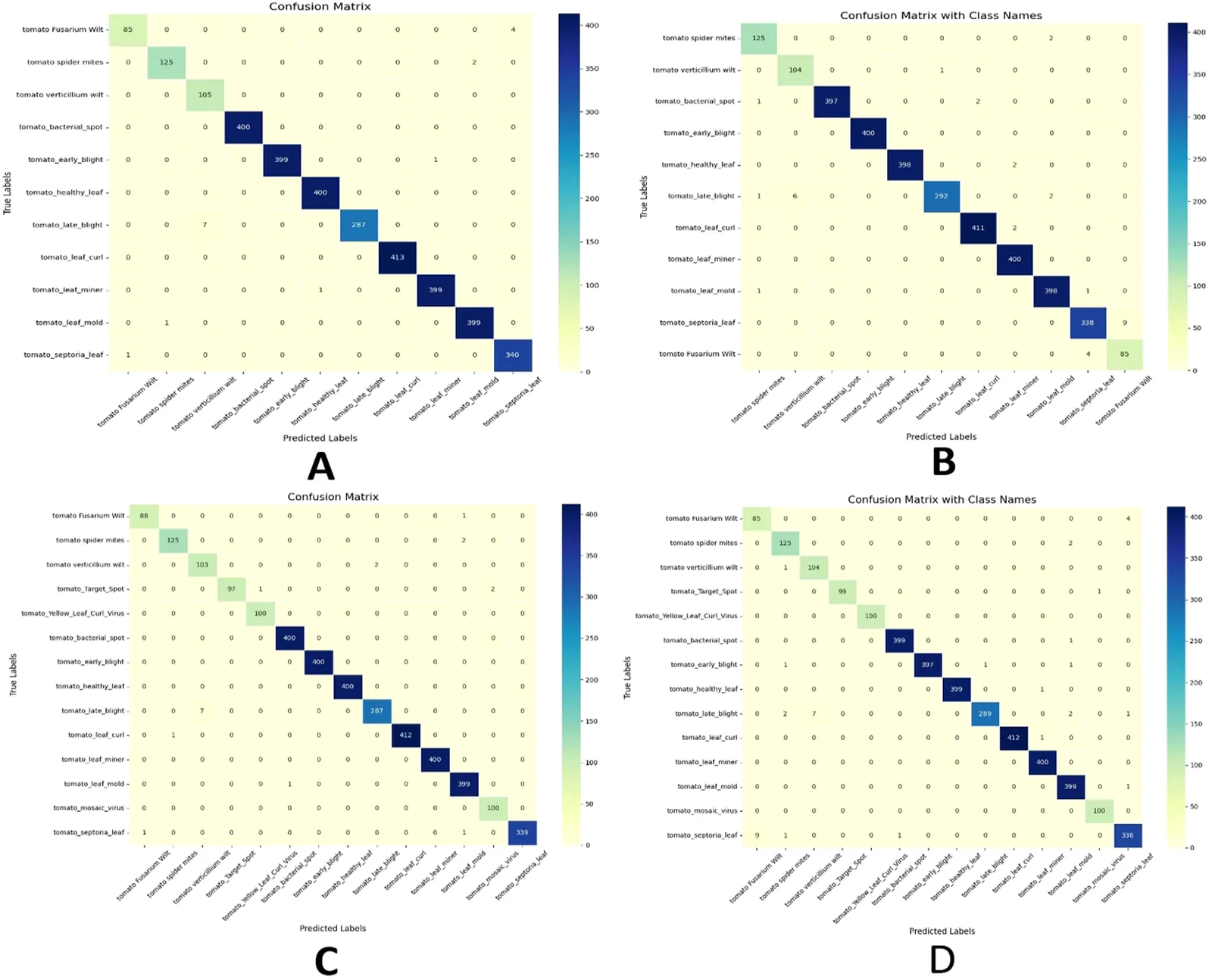
Confusion matrices of the proposed model: **(A)** 11-class PlantCity with GUBR; **(B)** 11-class PlantCity without GUBR; **(C)** 14-class combined dataset with GUBR; **(D)** 14-class combined dataset without GUBR.

**Figure 10:**
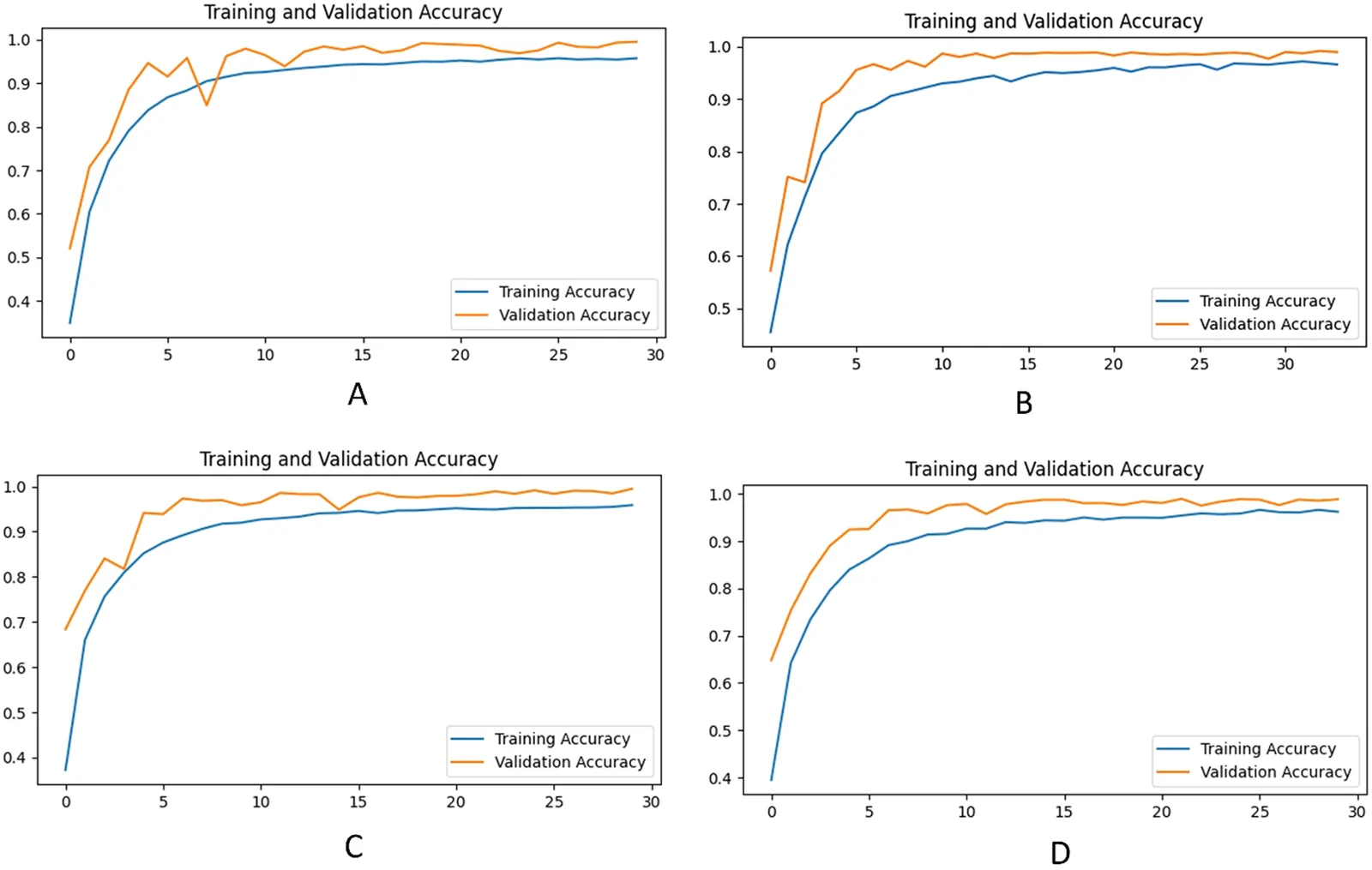
The training and validation accuracy of the proposed model: **(A)** 11-class with GUBR, **(B)** 11-class without GUBR, **(C)** 14-class with GUBR, and **(D)** 14-class without GUBR.

**Table 9:** Classification report of the proposed GSEBNet model.

Class	Precision	Recall	F1-score	Support
Tomato Fusarium Wilt	0.99	0.99	0.99	89
Tomato spider mites	0.99	0.98	0.99	127
Tomato verticillium wilt	0.94	0.98	0.96	105
Tomato Target Spot	1.00	0.97	0.98	100
tomato_Yellow_Leaf_Curl_Virus	0.99	1.00	1.00	100
Tomato bacterial spot	1.00	1.00	1.00	400
Tomato early blight	1.00	1.00	1.00	400
Tomato healthy leaf	1.00	1.00	1.00	400
Tomato late blight	0.99	0.98	0.98	294
Tomato leaf curl	1.00	1.00	1.00	413
Tomato leaf miner	1.00	1.00	1.00	400
Tomato leaf mold	0.99	1.00	0.99	400
Tomato mosaic virus	0.98	1.00	0.99	100
Tomato Septoria leaf	1.00	0.99	1.00	341

The results obtained by applying GUBR to different architectures (**Tables 3**, **4**) have been consistent, demonstrating that the contribution of GUBR is functional and task-specific, and not architectural or theoretical. Moreover, the experimental results shown in **Tables 3**, **4** confirm that GUBR consistently outperforms the classification performance of all the benchmark architectures (e.g., DenseNet121 + 0.30%, MobileNet +0.30%, and the proposed GSEBNet +0.30% for 11 classes; DenseNet121 + 0.93%, VGG16 + 2.26%, and GSEBNet +0.52% for 14 classes), thus proving its practical utility as a preprocessing module and not claiming any theoretical novelty.

The overall performance is good, but there are still some misclassifications. The most common misdiagnoses from the confusion matrices (**Figure 9**) are Early Blight and Target Spot, and Bacterial Spot and Septoria Leaf Spot. These errors are consistent with the difficulties of high intra-class variation and inter-class visual similarity mentioned in the Introduction. The lesions of these diseases are similar in color, texture, and morphological pattern, particularly under different light conditions and stages of disease development. Representative misclassified examples demonstrate that the model sometimes targets secondary symptoms or background areas instead of the primary lesion area. These residual errors suggest that there is still room for better fine-grained feature discrimination and domain robustness.

Moreover, **Figure 11** presents several representative predictions generated by the proposed model, illustrating its performance across diverse test cases. These examples highlight the model’s ability to accurately capture key patterns and produce high-quality outputs compared to the ground truth.

**Figure 11:**
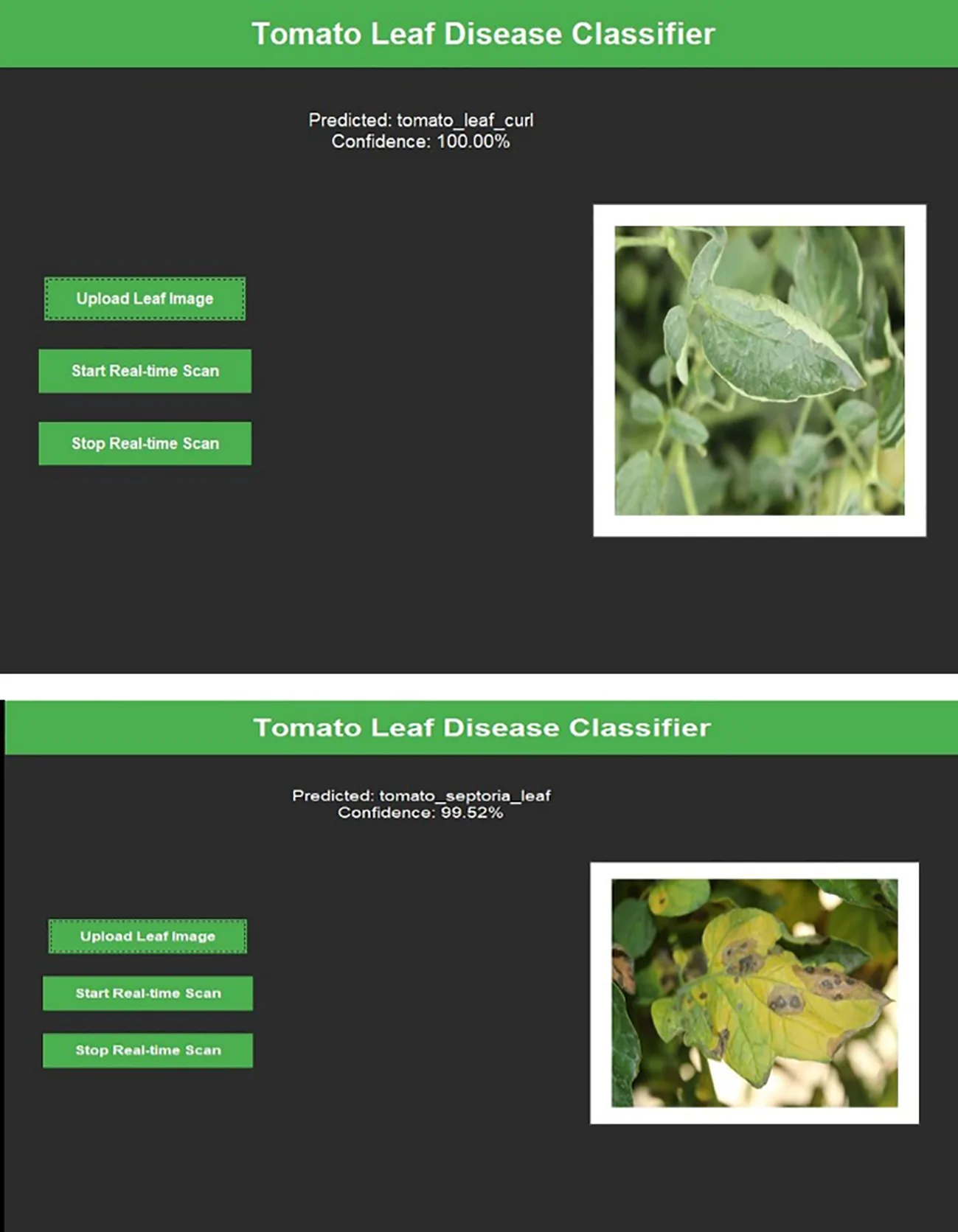
Sample prediction results using the proposed model.

### 4.2 XAI visualization

Three complementary explainable AI (XAI) methods, Grad-CAM, SHAP, and LIME, were used in addition to quantitative localization metrics to give a more complete and reliable interpretation of the model’s decision-making process. Grad-CAM identifies the discriminative regions the network focuses on, and SHAP and LIME independently validate that the attended regions are indeed the actual diseased tissue, not background artifacts. The Explanation Compactness Ratio, Foreground Concentration Ratio, and Peak-to-Foreground Hit Rate are further metrics that quantify the tightness and consistency of the model’s focus on the diseased region over the sample set.

The proposed GSEBNet is used to generate Grad-CAM attention maps as shown in **Figure 12**. The visualizations clearly show that the model is focused on the actual disease lesions and affected parts of the leaf and not on irrelevant background regions or healthy parts of the leaf. This is a confirmation of the effectiveness of the SE blocks embedded in the MBConv layers for learning discriminative disease-specific features.

**Figure 12:**
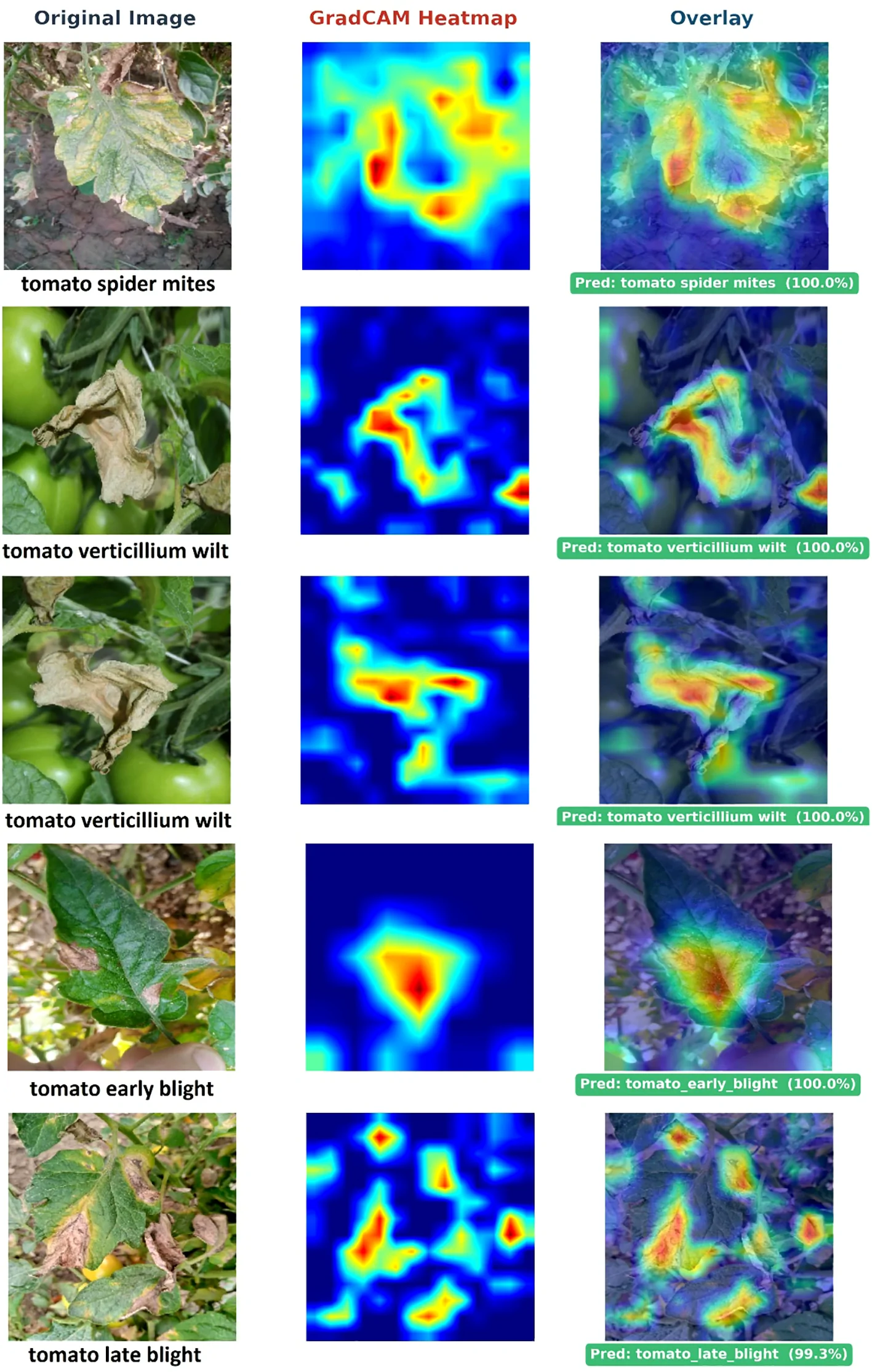
Grad-CAM visualization of GSEBNet for tomato leaf disease classification.

**Figure 13** shows explanations provided by SHAP for eight test samples across various disease classes and the healthy leaf class. The original image, the SHAP heatmap, and the overlay of the two are displayed side by side for each sample. The SHAP heatmap assigns a contribution score to each pixel, with brighter clustered points showing a higher positive contribution to the predicted class. In all the examples presented, the model achieves 100% prediction confidence, and the highlighted pixels always align with the lesion or the surface of the leaf, but not with the background or the hand holding the leaf.

**Figure 13:**
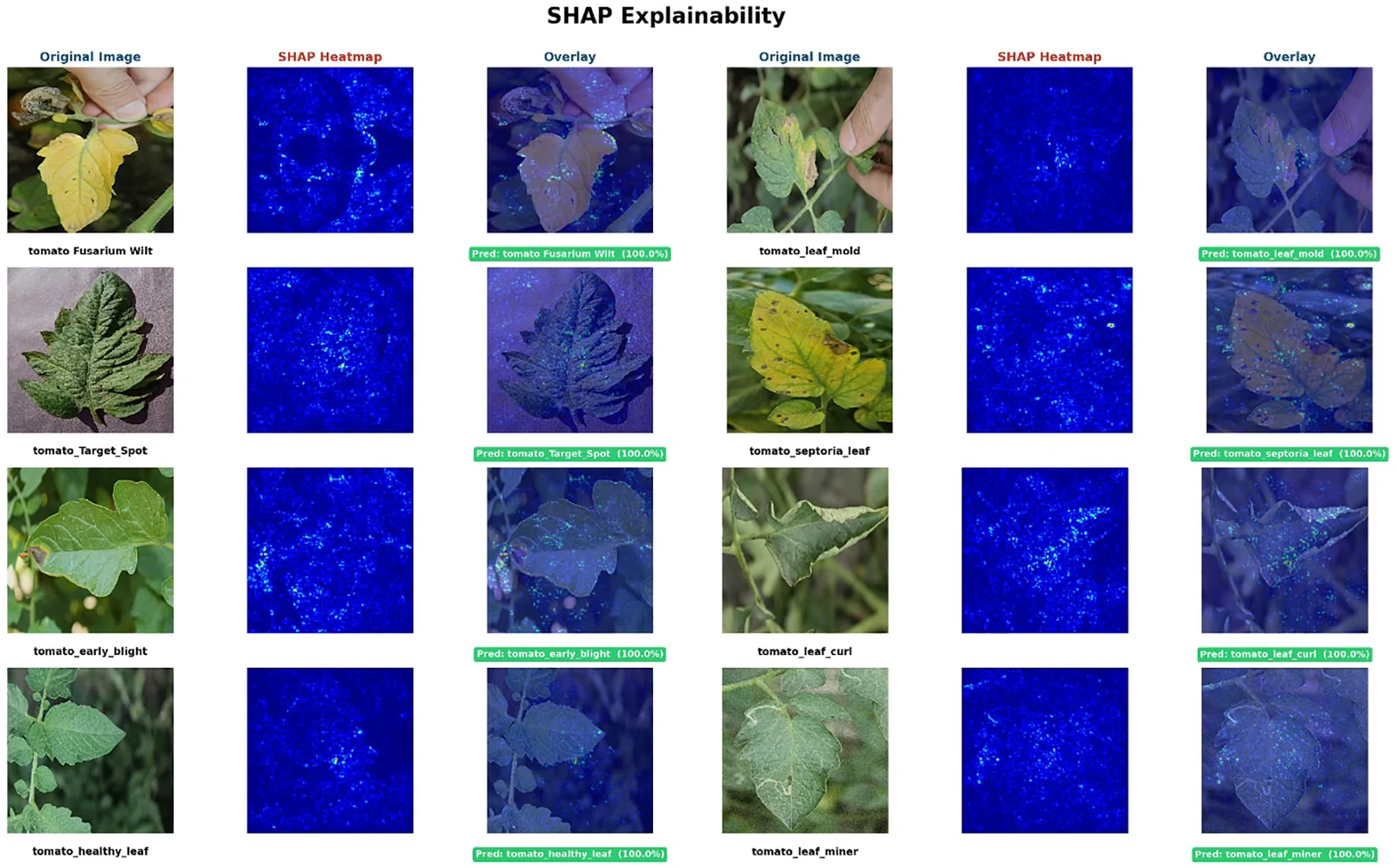
SHAP-based explainability heatmaps for representative tomato leaf samples, illustrating the pixel-level contributions driving individual model predictions.

**Figure 14** shows LIME-based explanations for four representative samples, including a healthy leaf case. The original image, the LIME super-pixel segmentation, and the image with the most influential region marked in red are shown for each sample. LIME is different from SHAP because it identifies a few contiguous super-pixel regions that are most important for the prediction of the label; this allows us to identify the specific patch of leaf tissue that is most important for the prediction. The regions outlined in each of the four cases represent areas of visibly diseased tissue or, in the case of the healthy sample, a representative area of undamaged leaf, and the model predictions are again correct with high confidence.

**Figure 14:**
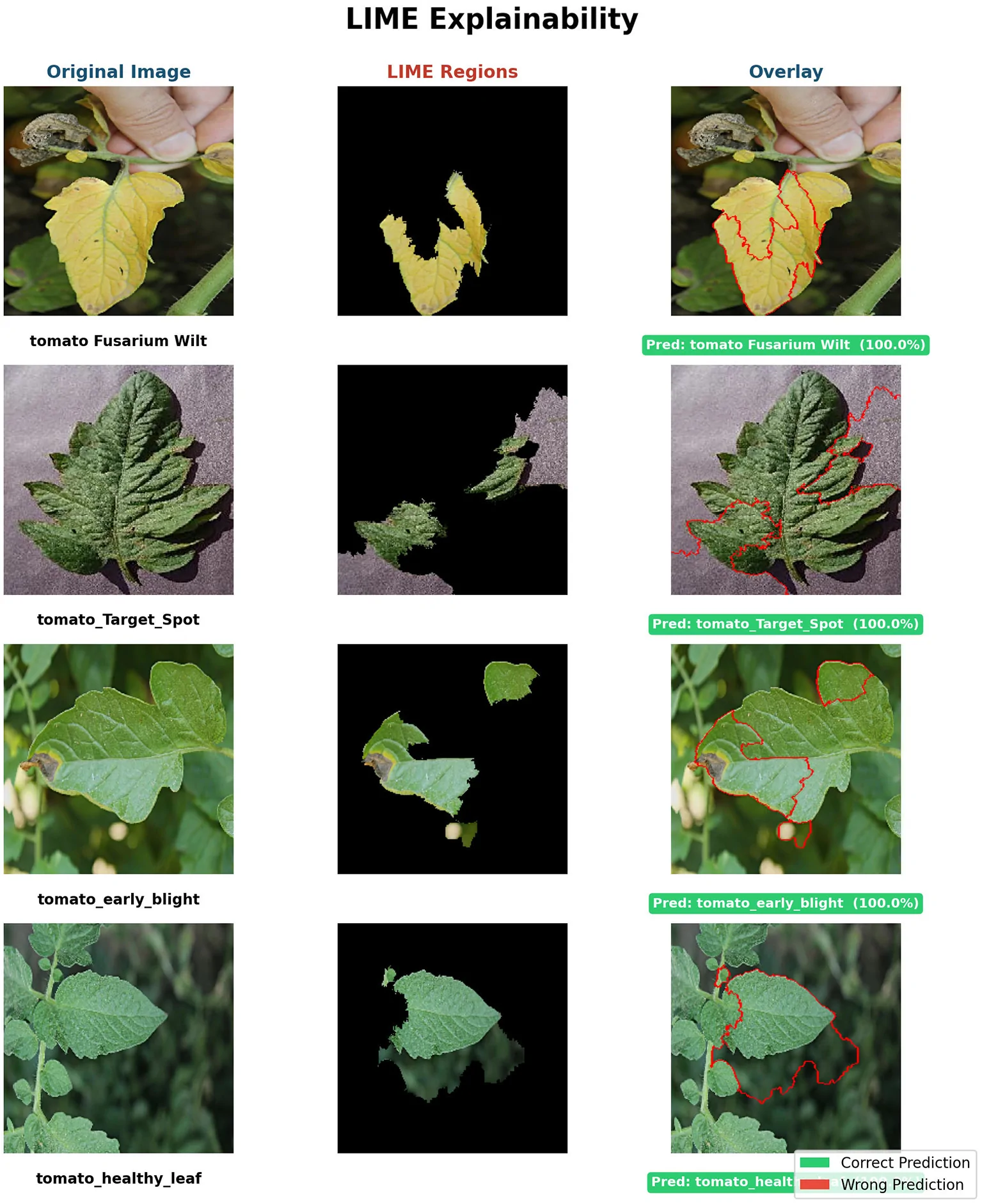
LIME-based explanation maps for representative tomato leaf samples, showing the local super-pixel regions that most influenced the predicted class.

To quantify the spatial precision of the Grad-CAM explanations, we calculate the Foreground Concentration Ratio (FCR), the Peak-to-Foreground Hit Rate (HIT), and the Compactness Ratio (CR) on the top 5% most activated region and its bounding box for six representative samples, as shown in **Figure 15**. The figure illustrates the original image, the Grad-CAM heatmap, the heatmap with the peak-activation point marked, and the top-5% activation region with the bounding box overlaid on the original image for each sample. The FCR values of most samples are around 0.99 with a HIT label, indicating that the peak activation occurs at the same location as the annotated diseased region, and the CR values range from approximately 0.24 to 0.75, suggesting that the compactness of the activated area is dependent on the size and shape of the underlying lesion. The yellow-leaf-curl-virus and bacterial-spot samples, however, have an FCR of 0.00, which means that for these two classes the maximum activation is not in the manually defined foreground area, although the correct classification was made.

**Figure 15:**
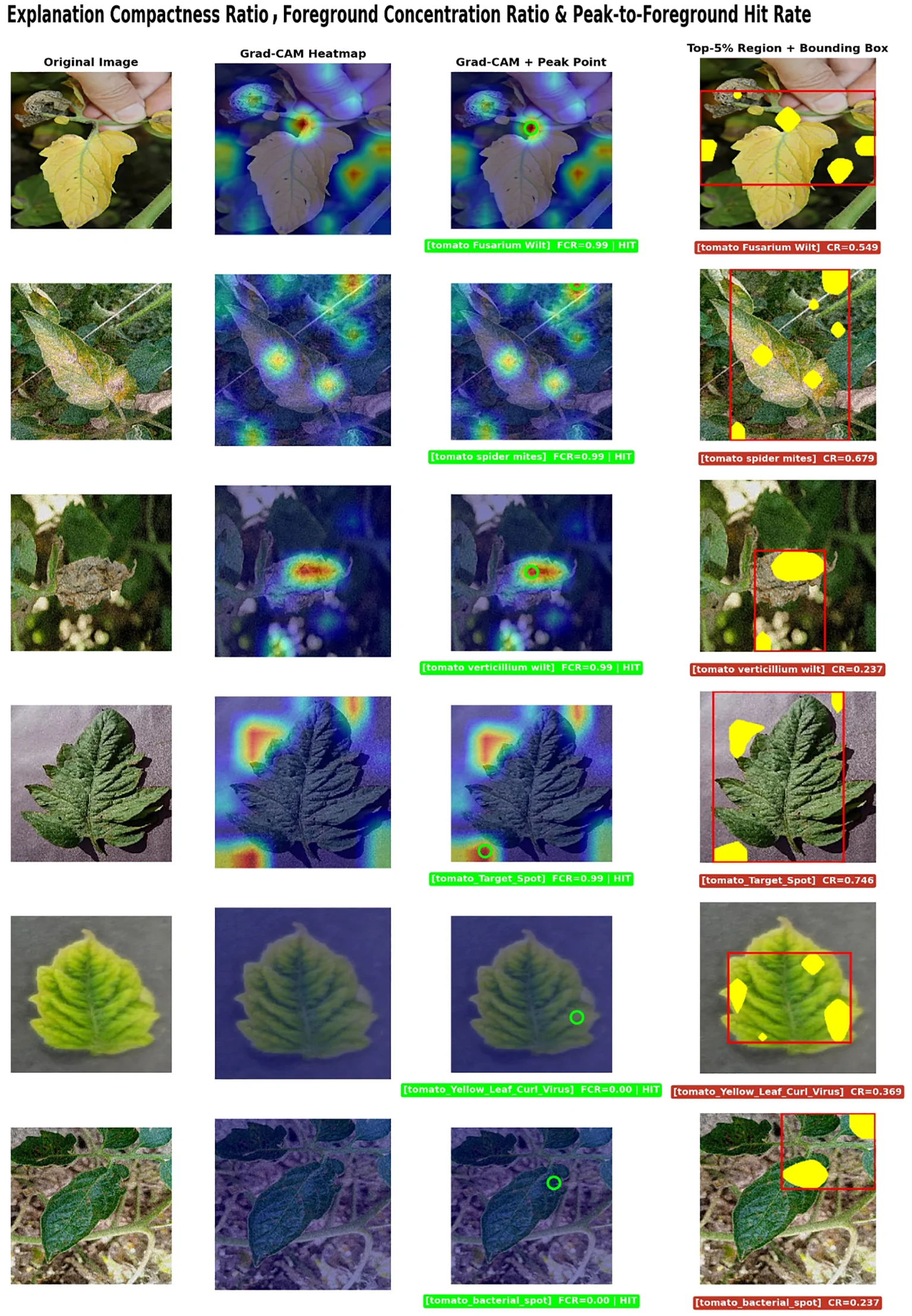
Explanation of compactness ratio, foreground concentration ratio, and peak-to-foreground hit rate analysis, quantifying the alignment between Grad-CAM attention and the annotated disease region across sample predictions.

The expanded LIME and SHAP visualizations (with examples from both PlantCity and PlantVillage) indicate that the model tends to focus on the areas of the leaf that are diseased, and not on the background or acquisition artifacts. The quantitative metrics in **Figure 15** show the Compactness Ratio, Foreground Concentration Ratio, and Peak-to-Foreground Hit Rate, and also confirm the correlation between the model’s attention and the annotated disease regions.

### 4.3 Ablation study

As can be seen in the ablation results reported in **Table 10**, the highest scores are obtained on both the 14-class and 11-class settings when using the full GSEBNet configuration (MBConv + SE blocks + GUBR). Each individual removal of a component (SE blocks, MBConv, or GUBR) results in a small but consistent performance drop (usually 0.1-0.5 percentage points). The differences observed are a few tenths of a percent, and no formal statistical significance test was performed, so we do not claim statistical significance. The results, however, show that the three components are complementary and that the full GSEBNet design provides the best overall performance.

**Table 10:** Ablation study of GSEBNet components on 14-class and 11-class configurations.

Configuration	Precision (%)	Recall (%)	F1-score	Accuracy (%)	Cohen’s Kappa
Full GSEBNet (MBConv + SE + GUBR) 14 classes	99.48	99.45	0.9946	99.48	0.9943
Full GSEBNet (MBConv + SE + GUBR) 11 classes	99.49	99.49	0.9949	99.50	0.9944
GSEBNet (MBConv only, without SE), 14 classes	99.40	99.40	0.9940	99.40	0.9934
GSEBNet (MBConv only, without SE and without GUBR), 14 classes	99.26	99.13	0.9920	99.26	0.9919
GSEBNet (SE only, without MBConv) + GUBR, 14 classes	99.40	99.35	0.9937	99.37	0.9931
GSEBNet (SE only, without MBConv and without GUBR), 14 classes	99.13	99.02	0.9907	99.10	0.9901
GSEBNet (MBConv only, without SE), 11 classes	99.08	99.05	0.9906	99.08	0.9897
GSEBNet (MBConv only, without SE and without GUBR), 11 classes	99.11	98.96	0.9903	99.05	0.9894
GSEBNet (SE only, without MBConv) + GUBR, 11 classes	99.41	99.35	0.9938	99.38	0.9930
GSEBNet (SE only, without MBConv and without GUBR), 11 classes	98.90	98.84	0.9887	98.87	0.9874

The individual contribution of each GUBR operation was evaluated on the 14-class combined dataset. The performance of the main preprocessing components and their combinations is reported in **Table 11**. Brightness reduction alone and Brightness reduction + Unsharp masking resulted in the best scores for the tested variants, whereas Gamma correction alone yielded relatively poor performance. These results show that the sequential combination of operations is beneficial, but the complete 3-stage GUBR pipeline is the best configuration when combined with the complete GSEBNet architecture.

**Table 11:** GUBR component-level ablation on the 14-class dataset.

Experiment	Accuracy	Precision	Recall	F1-score
Gamma correction + Unsharp masking	98.37% ± 0.21%	97.34% ± 0.36%	97.02% ± 0.41%	0.9718 ± 0.0037
Brightness reduction only (beta = 0.9)	99.24% ± 0.14%	98.75% ± 0.26%	98.75% ± 0.27%	0.9875 ± 0.0025
Gamma correction only (γ = 0.8)	97.74% ± 0.29%	97.26% ± 0.41%	97.84% ± 0.31%	0.9748 ± 0.0036
Gamma correction + Brightness reduction	98.91% ± 0.17%	98.57% ± 0.26%	98.39% ± 0.30%	0.9848 ± 0.0026
Unsharp masking + Brightness reduction	99.13% ± 0.16%	98.58% ± 0.28%	98.61% ± 0.29%	0.9860 ± 0.0027
Unsharp masking only	98.93% ± 0.17%	98.09% ± 0.32%	98.06% ± 0.34%	0.9808 ± 0.0032

To assess the stability of the proposed model, stratified 5-fold cross-validation was performed on the 14-class dataset. The accuracy, precision, recall, and F1-score for each fold are given in **Table 8**. The performance of GSEBNet is very consistent across all folds, with only slight variations, which is a good indicator of the stability of the strong performance of GSEBNet across different data partitions.

The per-fold confusion matrices of the 5-fold cross-validation are presented in **Figures 16**–**18**. The results for Fold-1 and Fold-2 are shown in **Figure 16**, Fold-3 and Fold-4 in **Figure 17**, and Fold-5 in **Figure 18**. For all folds, the majority of classes are classified with very high accuracy, and only a few confusions are left, as reported in the quantitative metrics in **Table 8**.

**Figure 16:**
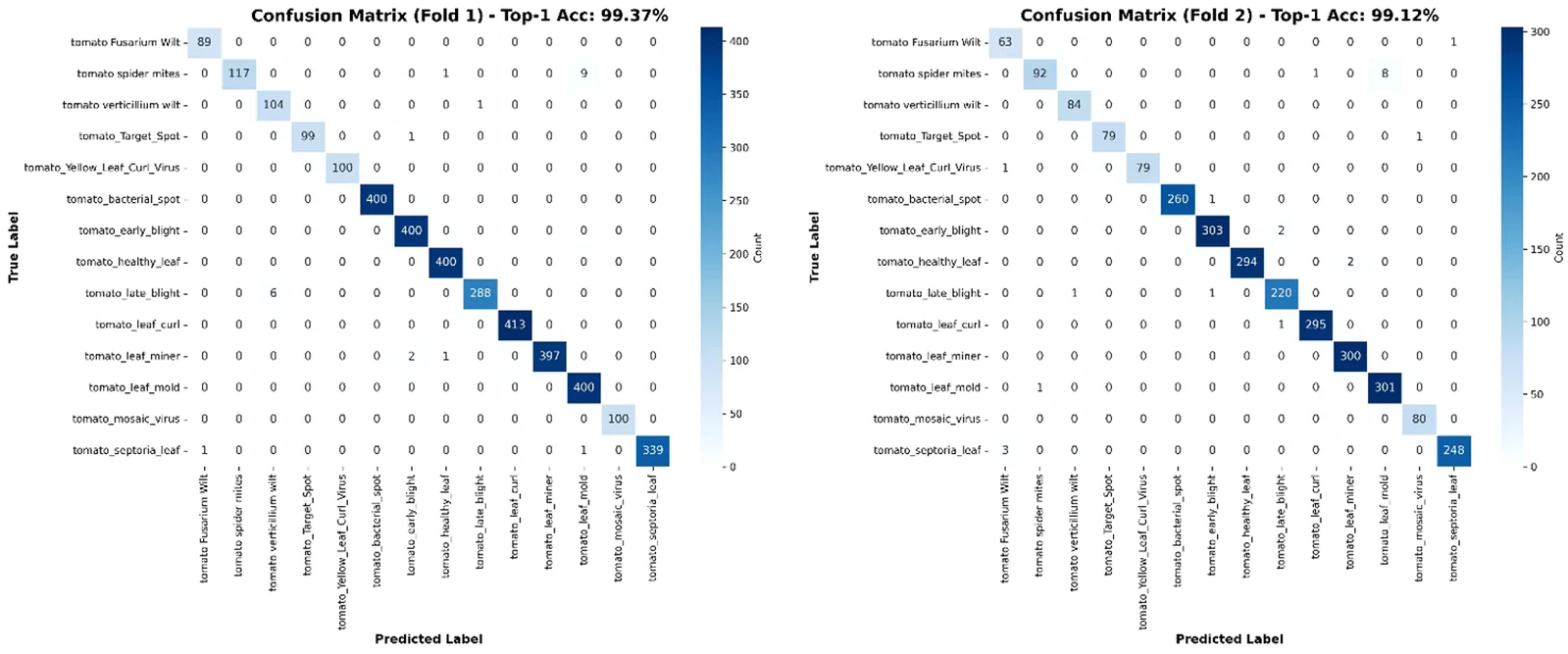
Confusion matrices of GSEBNet for Fold-1 and Fold-2 on the 14-class dataset.

**Figure 17:**
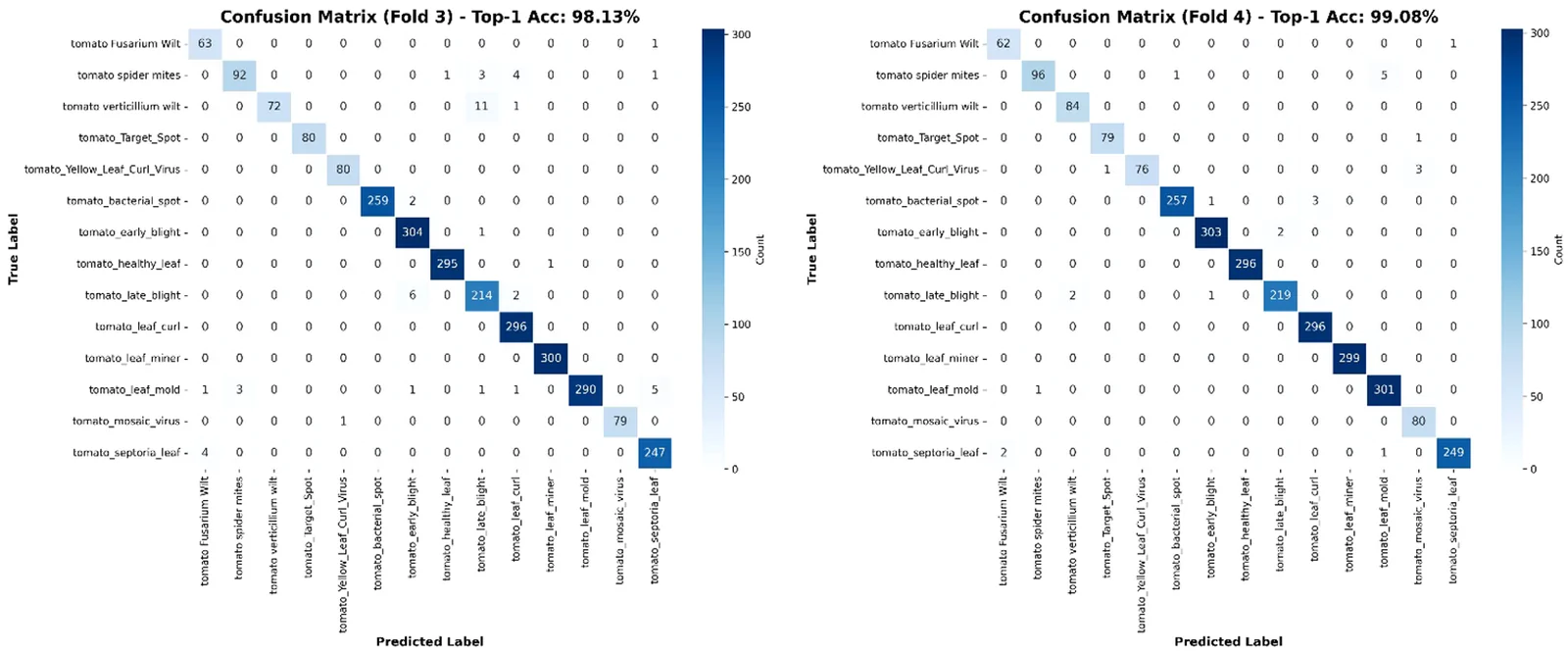
Confusion matrices of GSEBNet for Fold-3 and Fold-4 on the 14-class dataset.

**Figure 18:**
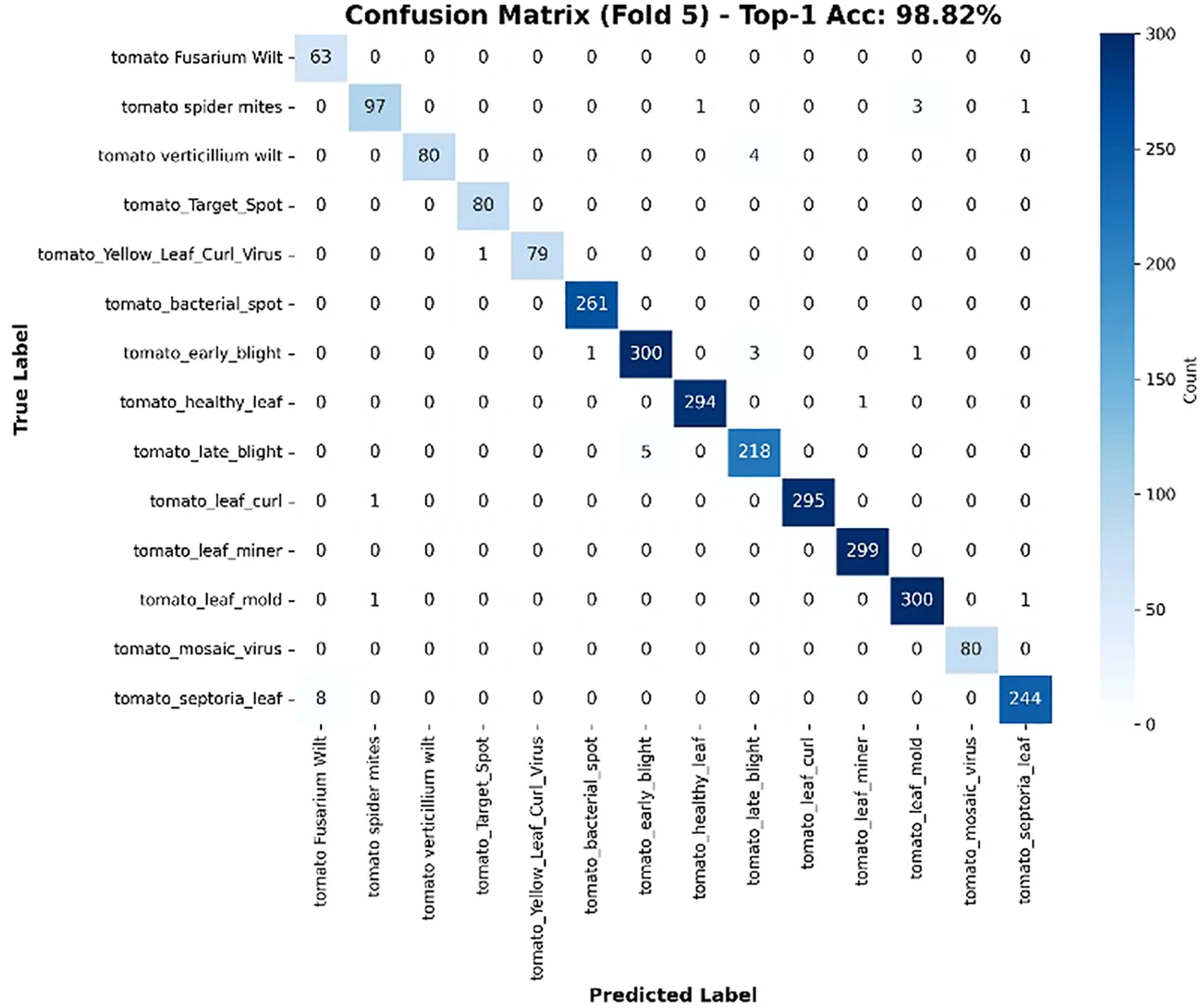
Confusion matrix of GSEBNet for Fold-5 on the 14-class dataset.

The contribution of the GUBR preprocessing pipeline was further analyzed at the component level as shown in **Table 11**. All results are presented as mean ± standard deviation across 5-fold cross-validation, stratified by class. The best performance is obtained with the full GUBR sequence (Gamma + Unsharp + Brightness), and the partial combinations of these operations or single operations score lower. This means that the three operations, performed in sequence, are more effective than any of the three conventional preprocessing operations.

Furthermore, **Table 8** shows the overall performance of the full GSEBNet model on the 14-class dataset using 5-fold cross-validation, which demonstrates the consistency of the reported results with respect to different partitions of the data.

**Table 7** compares the complexity and inference speed of the proposed GSEBNet with DenseNet121, DenseNet169, DenseNet201, MobileNet, MobileNetV2, VGG16, and VGG19 to assess the deployment feasibility of the proposed GSEBNet. The 0.48M parameters (about 4.7× fewer than MobileNetV2 and 41.7× fewer than VGG19) and 3.84 MB of storage space make GSEBNet the lightest network in the comparison. Note that the reported inference time of 69.79 ms per image was measured on an RTX 2060 Super GPU and not on actual mobile or agricultural edge devices (e.g., Raspberry Pi, smartphone, or Jetson). It has a lower computational cost of 1.15 GFLOPs than the DenseNet and VGG families, and it has a similar inference time of 69.79 ms per image (14.3 FPS) to the MobileNet family, but is much faster than all DenseNet and VGG variants. The results show that GSEBNet provides the best balance between model size and inference time, making it a promising candidate for future deployment on resource-limited devices.

To address the potential source-confounding issue arising from the mixed origin of the 14-class dataset, cross-domain experiments were performed (**Table 12**). When GSEBNet was trained on PlantCity and tested on PlantVillage, accuracy dropped to 27.88%; in the reverse direction, the accuracy fell further to 14.82%. These results demonstrate that the very high performance (99.48%) obtained on the combined dataset is strictly in-domain. The model currently fails to generalize across different acquisition conditions and is highly sensitive to dataset-specific characteristics such as background, illumination, and image quality. Consequently, the strong in-domain results should not be interpreted as evidence of robustness under diverse real-world imaging conditions.

**Table 12:** Performance comparison of GSEBNet (with and without GUBR) on in-domain and cross-domain settings.

Experiment	Train dataset	Test dataset	Accuracy	Precision	Recall	F1-score
Cross-Domain 1 (GSEBNet without GUBR)	PlantVillage (10 classes)	PlantVillage	89.83%	90.43%	89.39%	0.8991
GSEBNet with GUBR	PlantVillage (10 classes)	PlantVillage	92.34%	92.82%	91.96%	0.9238
Cross-Domain 2	PlantCity	PlantVilage	27.88%	28.17%	27.39%	0.2777
	PlantVilage	PlantCity	14.82%	15.07%	14.44%	0.1475

**Table 13** presents a performance comparison of the proposed GSEBNet model against several recent state-of-the-art works. The results demonstrate that GSEBNet achieves highly competitive accuracy, obtaining 99.50% on 11 classes and 99.48% on 14 classes. These results either outperform or remain comparable to existing approaches, highlighting the effectiveness and robustness of the proposed method across varying classification complexities.

**Table 13:** Presents a performance comparison of the proposed GSEBNet model against several recent studies.

Existing work	Classification classes	Accuracy
(Al-Gaashani et al., 2022)	6	97%
(Zhao et al., 2021)	10	96.81%
(Zhou et al., 2021)	9	95%
(Islam et al., 2022)	7	98%
(Zhang et al., 2022)	7	98.9%
(Chen et al., 2022)	10	98%
(Ahmed et al., 2022)	10	99.30%
(Sanida et al., 2023)	10	99.17%
(Javidan et al., 2024)	7	95.58%
(Thangaraj et al., 2024)	10	99.61%
(Alnamoly et al., 2024)	10	99.10%
(Khan et al., 2024)	10	98.52%
(Abouelmagd et al., 2024)	10	96.39%
(Paul et al., 2023)	11	99.11%
(Peyal et al., 2023)	11	95%
(Yildiz et al., 2024)	5	96%
**Proposed Work**	**11**	**99.50%**
**Proposed Work**	**14**	**99.48%**

Direct numerical comparison should be interpreted with caution because the listed studies used different datasets and experimental protocols.

The results show that GSEBNet achieves highly competitive accuracy (99.50% on 11 classes and 99.48% on 14 classes). However, because the listed methods were evaluated on different datasets, class counts, and experimental protocols, these numerical differences should be interpreted with caution and do not constitute a direct claim of superiority under identical conditions. Bold values indicate the best performance results achieved by the proposed GSEBNet model.

## 5 Conclusion

In this study, we introduced a specialized architectural framework that combines CNNs with MBConv and SE blocks together with a domain-specific pre-processing pipeline, GUBR. Although MBConv, SE blocks, gamma correction, and unsharp masking are established techniques, the contribution of this work resides in their task-specific integration and co-design for the particular challenges of real-field tomato leaf disease imagery. The proposed framework is not based on the novelty of individual operators, but rather on task-specific optimization: the custom depth-width progression and SE placement in GSEBNet are tailored to capture subtle, visually similar disease patterns typical of tomato leaf imagery, while the GUBR pipeline is specifically parameterized to mitigate degradation patterns (uneven illumination, motion blur, and low contrast) common in field-captured agricultural datasets. This synergistic design allows for strong feature learning and inference-efficient design, as demonstrated by the empirical results that outperform general-purpose architectures. We made significant progress by developing a precise model that effectively identifies diseases in tomato leaves. Our models are trained to classify tomato leaves as healthy or diseased from visual input. The proposed system processes the input image in a short time and gives the result to the user in real time for further action. The innovation has the potential to transform the early detection of disease in tomato crops by utilizing technologies such as mobile devices, computers, and automated equipment. We used accuracy, precision, recall, Cohen’s kappa, and F1 score to assess the performance of our model. The results are outstanding. The test accuracy was 99.48% on the 14-class combined dataset and 99.50% on the 11-class PlantCity subset. Specifically, the model was able to accurately identify diseases with a precision of 99.49% in 11 classes and 99.48% in 14 classes. The F1 scores were almost perfect (0.99). Recall was perfect (0.99) for all 14 and 11 classes. Cohen’s kappa coefficient showed a high level of agreement (0.9927). The confusion matrix showed that our model achieved 100% accuracy in 50% of the test dataset classes and 99% accuracy in the remaining classes. This performance surpasses many methods currently proposed in the literature.

### 5.1 Limitations

While the in-domain results are good, there are a number of limitations to be noted. First, the 14-class dataset is a combination of images from two different sources (PlantCity and PlantVillage), which may introduce a source/domain bias. Second, the data split was done at the image level instead of the plant level, meaning that there is a residual risk of near-duplicate leakage. Third, hyperparameters were manually chosen for validation performance and not by an exhaustive automated search. Fourth, all measurements are done at inference time, which is not the same as real-world latency on resource-constrained edge devices like Raspberry Pi, smartphones, or Jetson boards. Most importantly, the significant performance degradation in the cross-domain experiments (**Table 8**) shows that robustness to large domain shifts between field-captured and lab-acquired images is not yet a solved problem of the current framework, but rather an issue that needs to be addressed.

### 5.2 Future work

Future work should be directed towards using the model in real agricultural environments and towards mitigating the source-confounding/domain-shift issue that was found between PlantCity and PlantVillage. We want to increase the robustness by adding more multi-source data, domain-adaptation techniques, and more classes and crop species. Furthermore, a rigorous plant-level and site-level data splitting protocol will be used in the expanded PlantCity benchmark to completely remove the near-duplicate leakage risk and to offer more robust evidence of generalization.

## Data Availability

The PlantCity dataset generated and analyzed in this study is publicly available at: https://data.mendeley.com/datasets/w8kh2xkspx/4, and the PlantVillage dataset used in this study is publicly available at: https://data.mendeley.com/datasets/tywbtsjrjv/1.
